# Supplementary figures and images for: Improved digital chest tomosynthesis image quality by use of a projection-based dual-energy virtual monochromatic convolutional neural network with super resolution
Source: PLoS One. 2020 Dec 31;15(12):e0244745. doi: 10.1371/journal.pone.0244745 (PMC7774945; doi:10.1371/journal.pone.0244745)

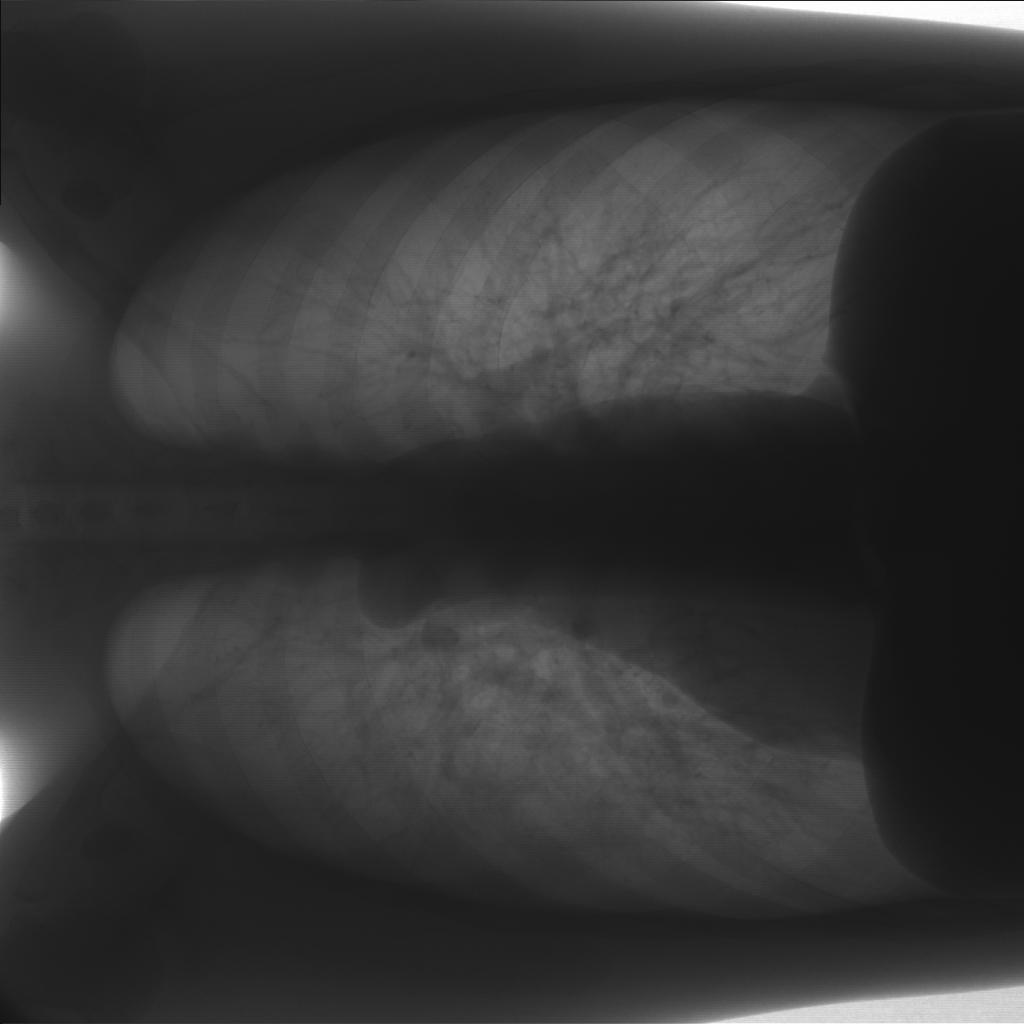

Supplement: S1 File — (ZIP) [file pone.0244745.s001.zip › Figure_1/Fc.jpg]

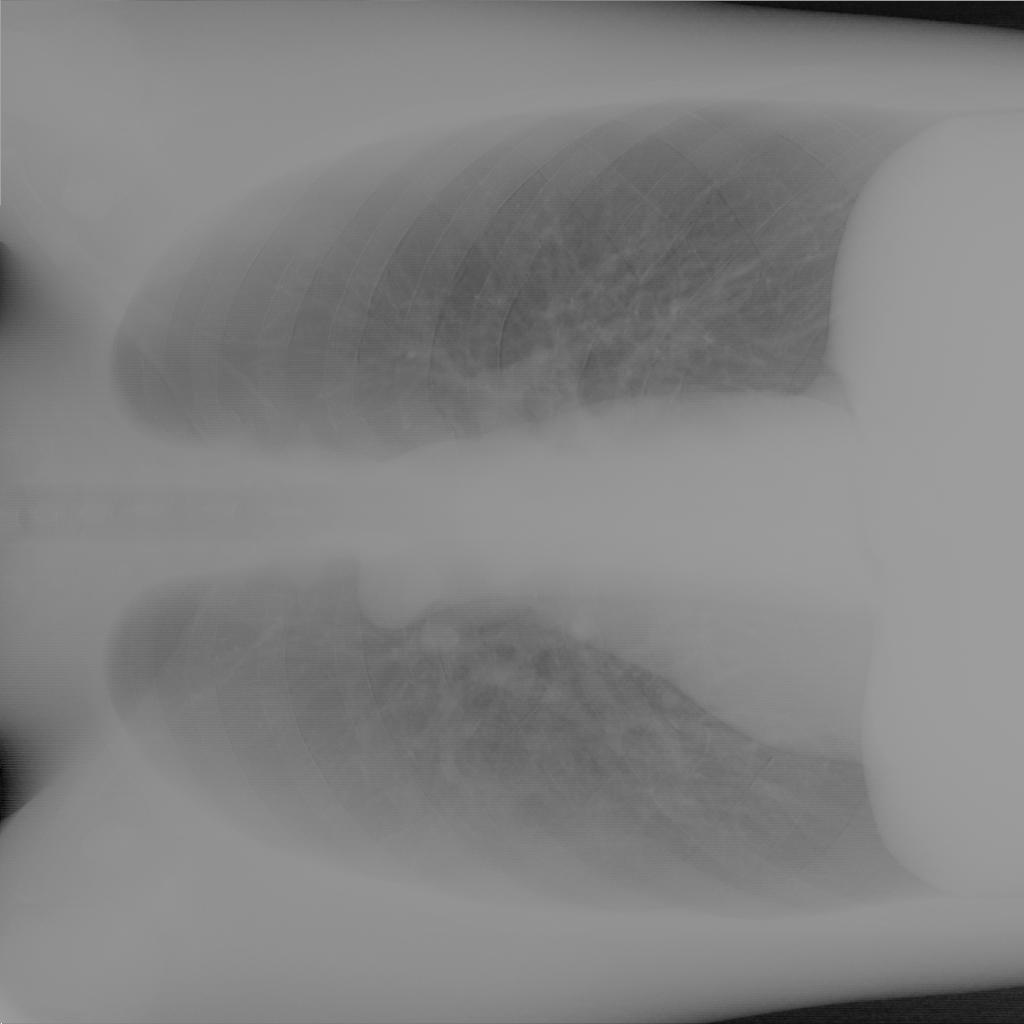

Supplement: S1 File — (ZIP) [file pone.0244745.s001.zip › Figure_1/Fp.jpg]

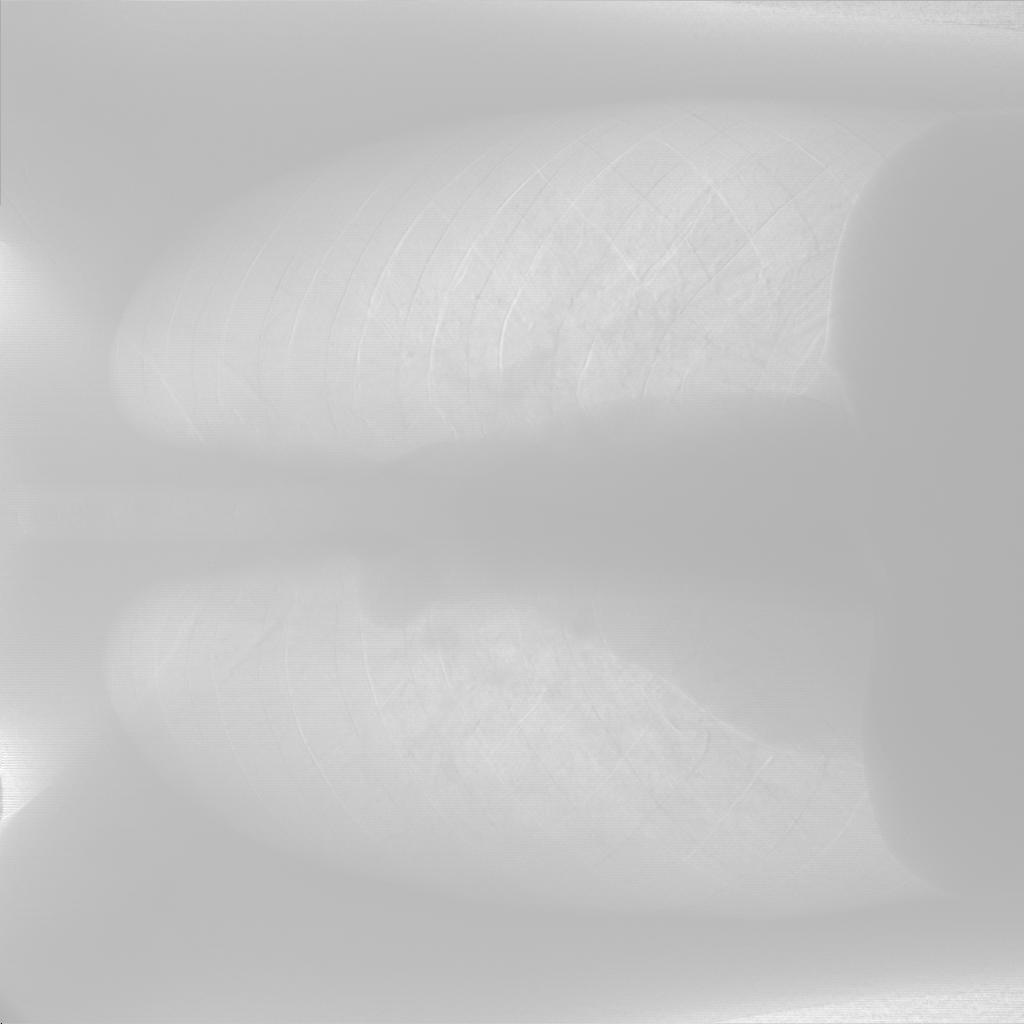

Supplement: S1 File — (ZIP) [file pone.0244745.s001.zip › Figure_1/Fu.jpg]

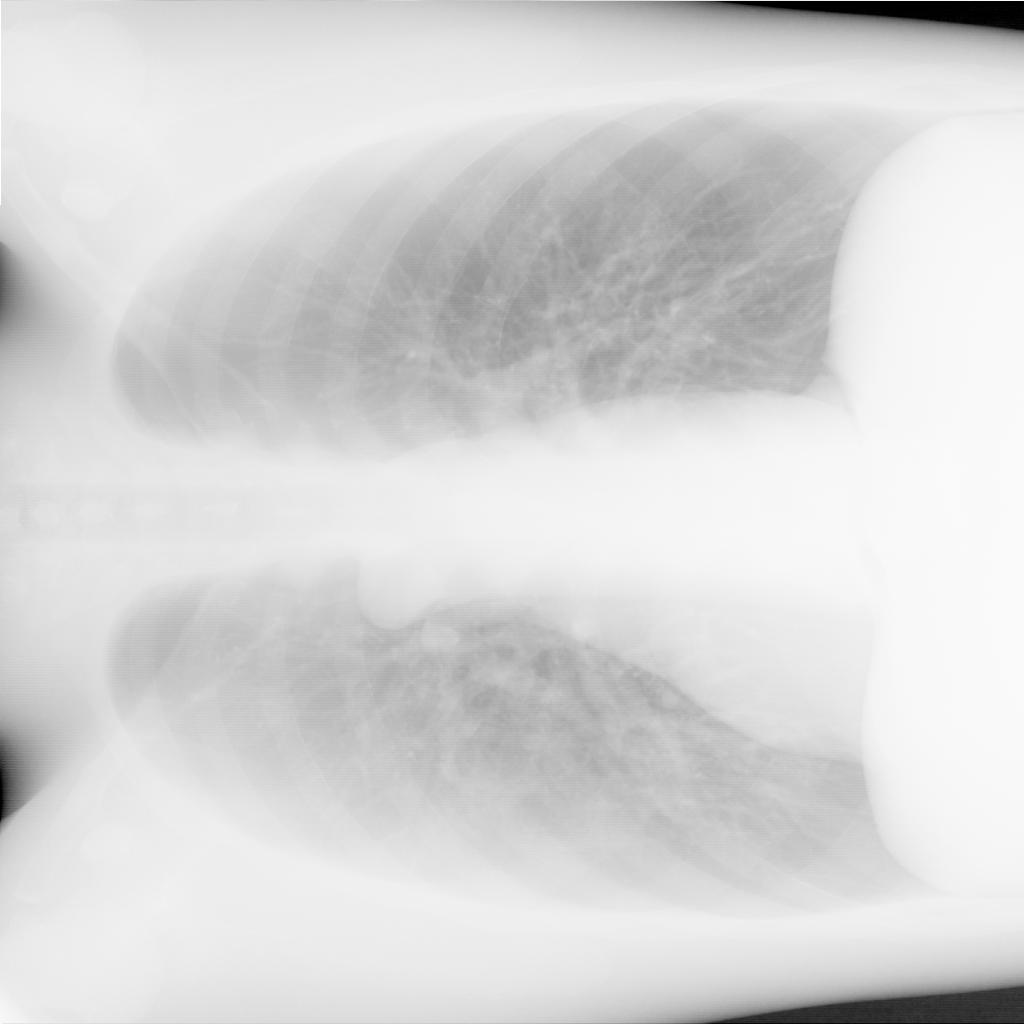

Supplement: S1 File — (ZIP) [file pone.0244745.s001.zip › Figure_1/High-kV(IH).jpg]

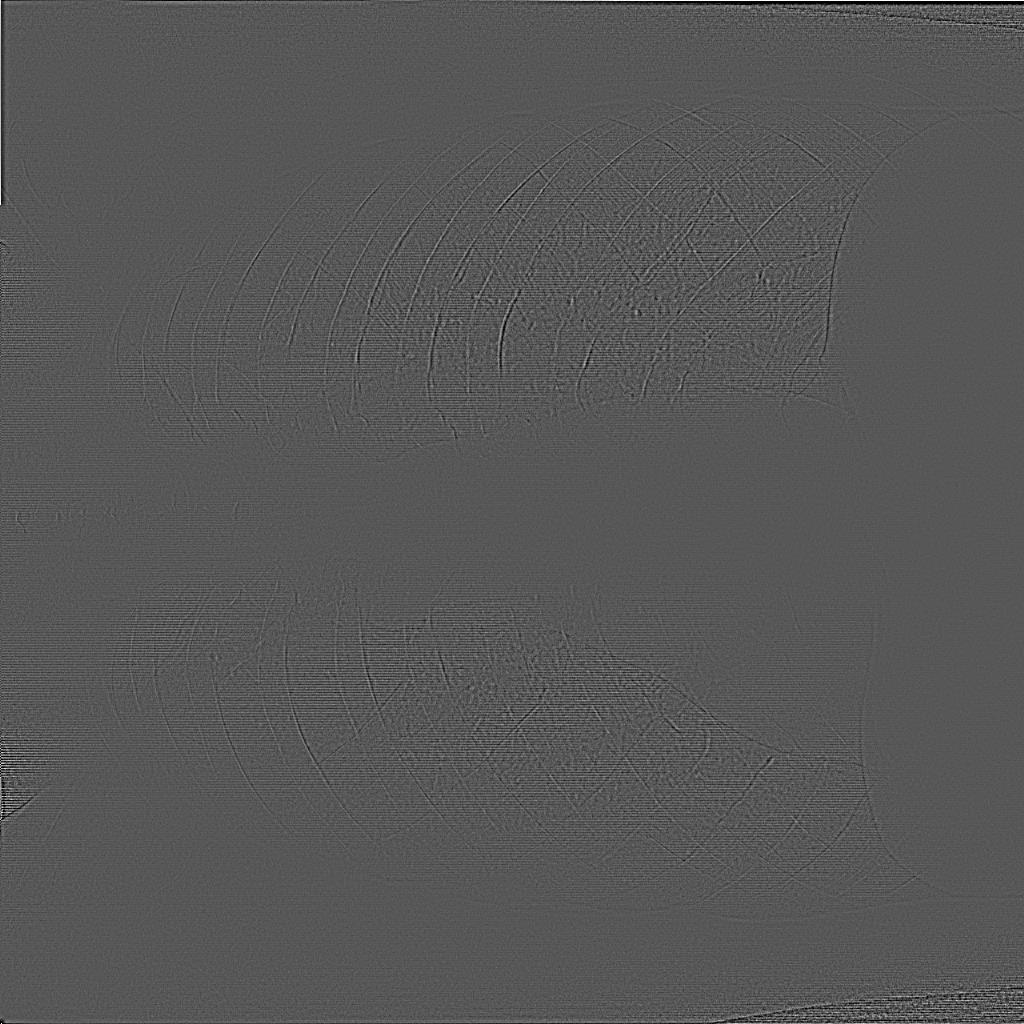

Supplement: S1 File — (ZIP) [file pone.0244745.s001.zip › Figure_1/HR(G).jpg]

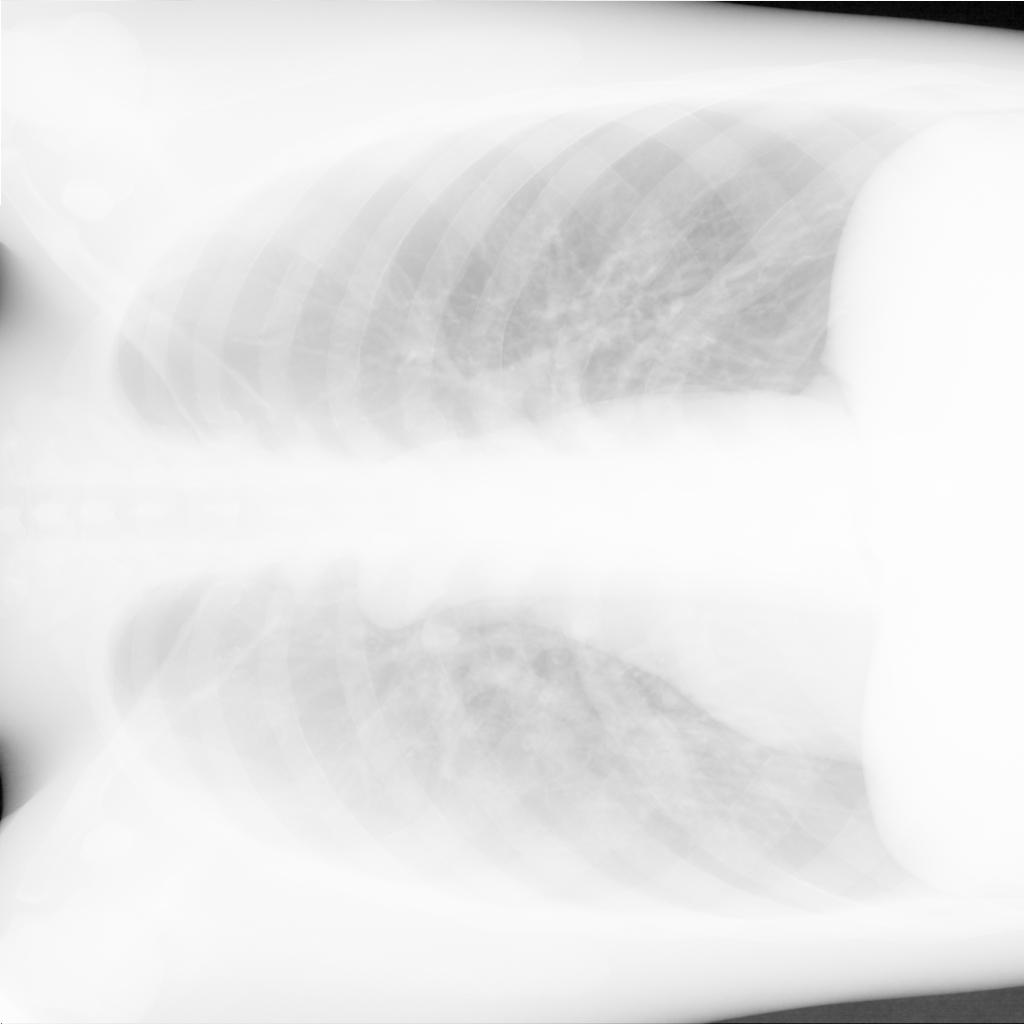

Supplement: S1 File — (ZIP) [file pone.0244745.s001.zip › Figure_1/Low-kV(IL).jpg]

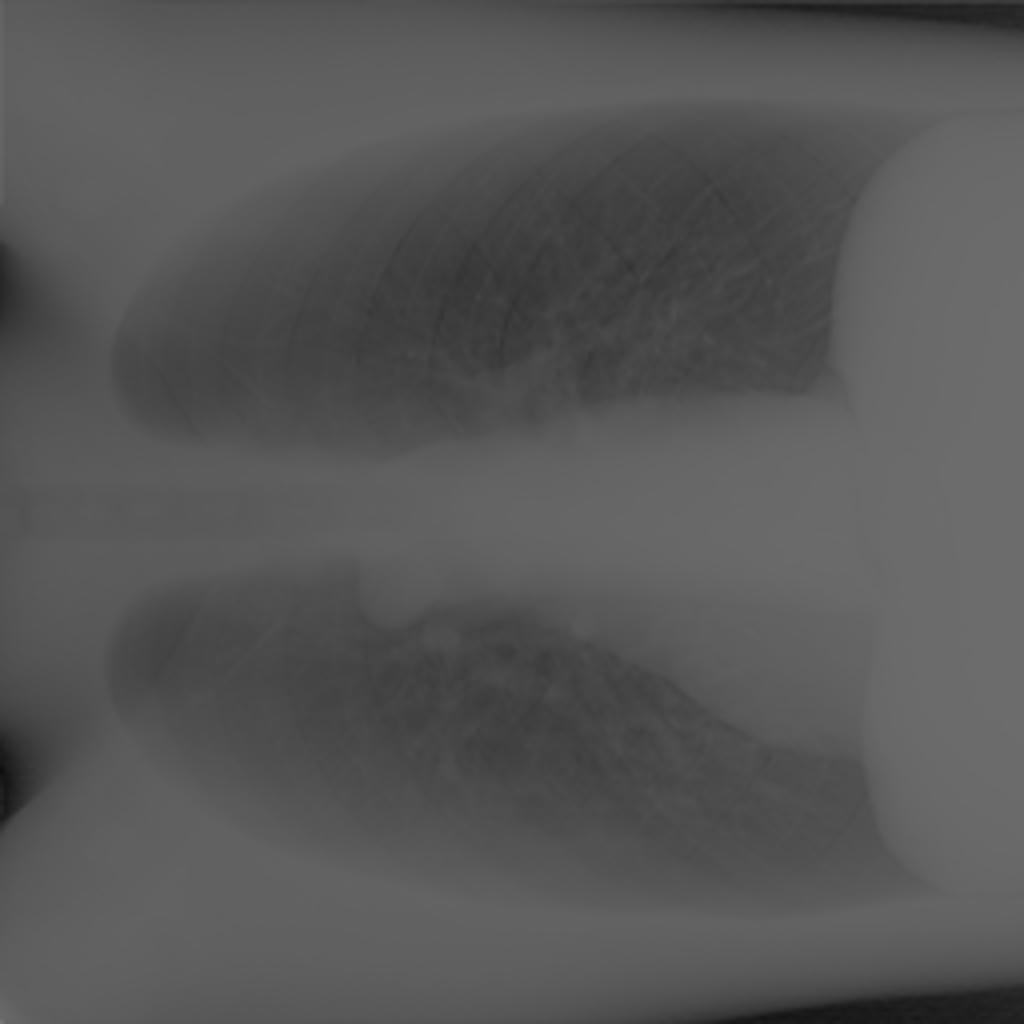

Supplement: S1 File — (ZIP) [file pone.0244745.s001.zip › Figure_1/LR(H).jpg]

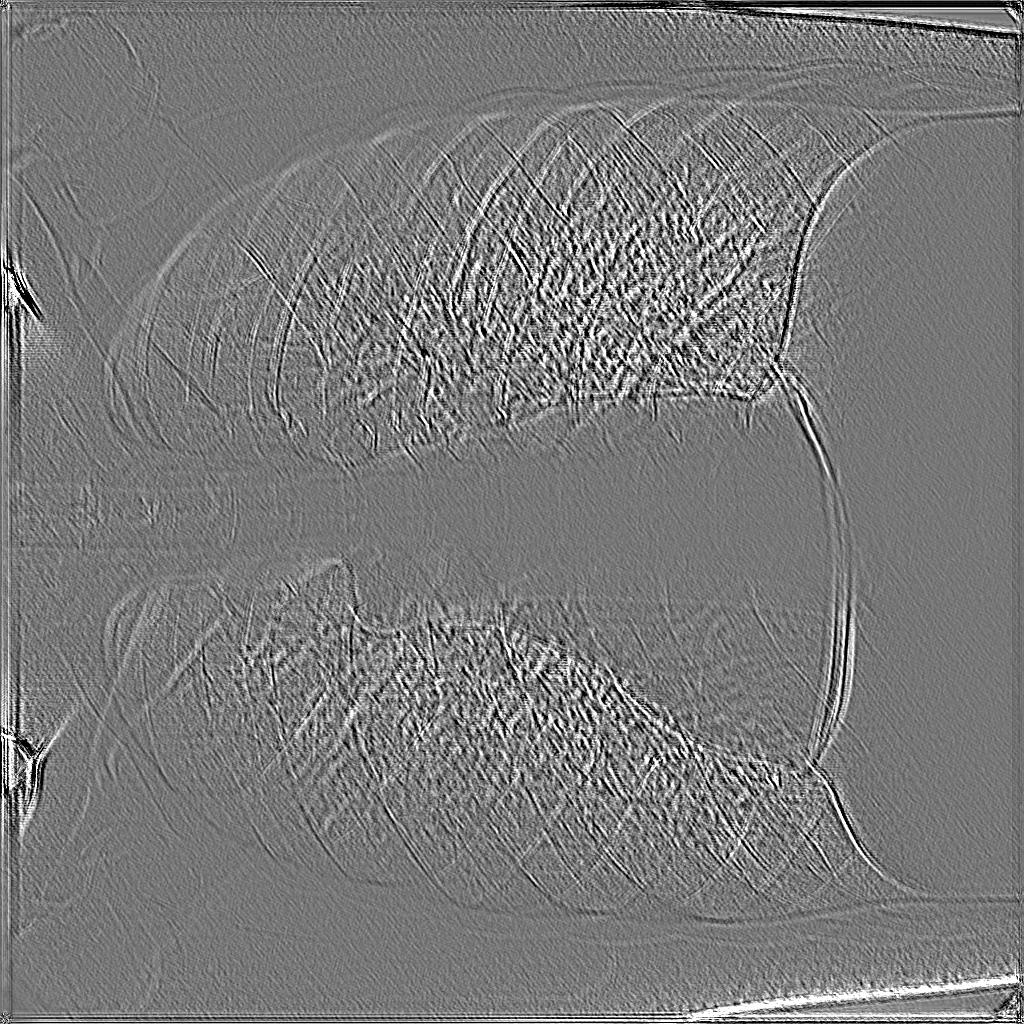

Supplement: S1 File — (ZIP) [file pone.0244745.s001.zip › Figure_1/residual(r).jpg]

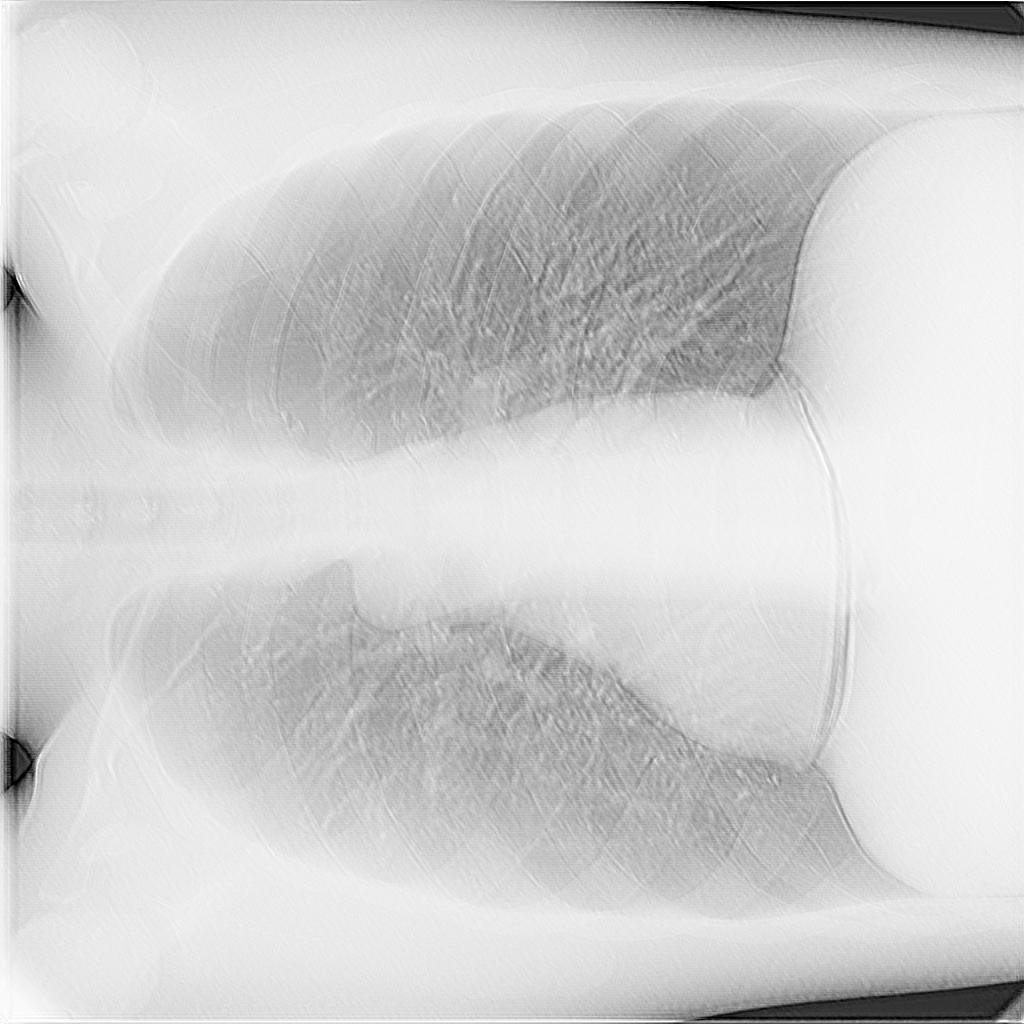

Supplement: S1 File — (ZIP) [file pone.0244745.s001.zip › Figure_1/SR(G).jpg]

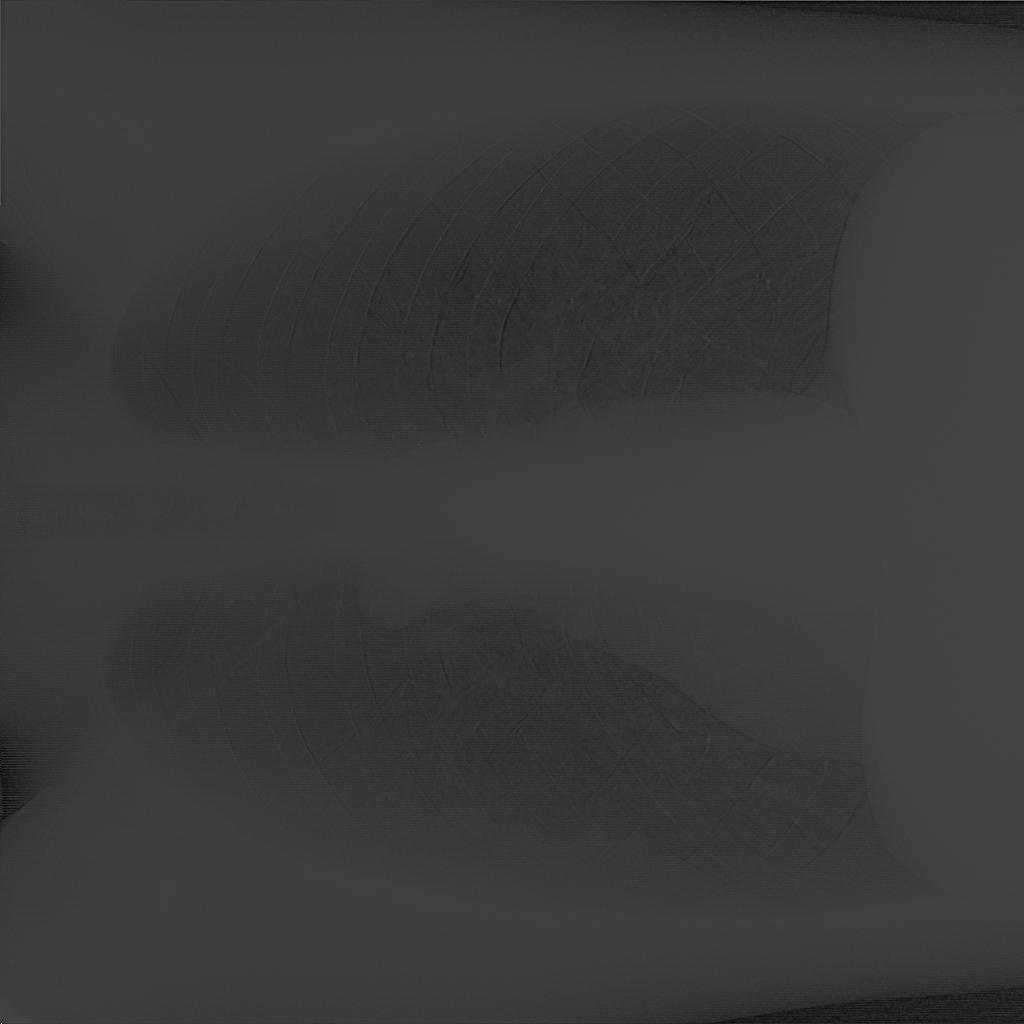

Supplement: S1 File — (ZIP) [file pone.0244745.s001.zip › Figure_1/UM(H).jpg]

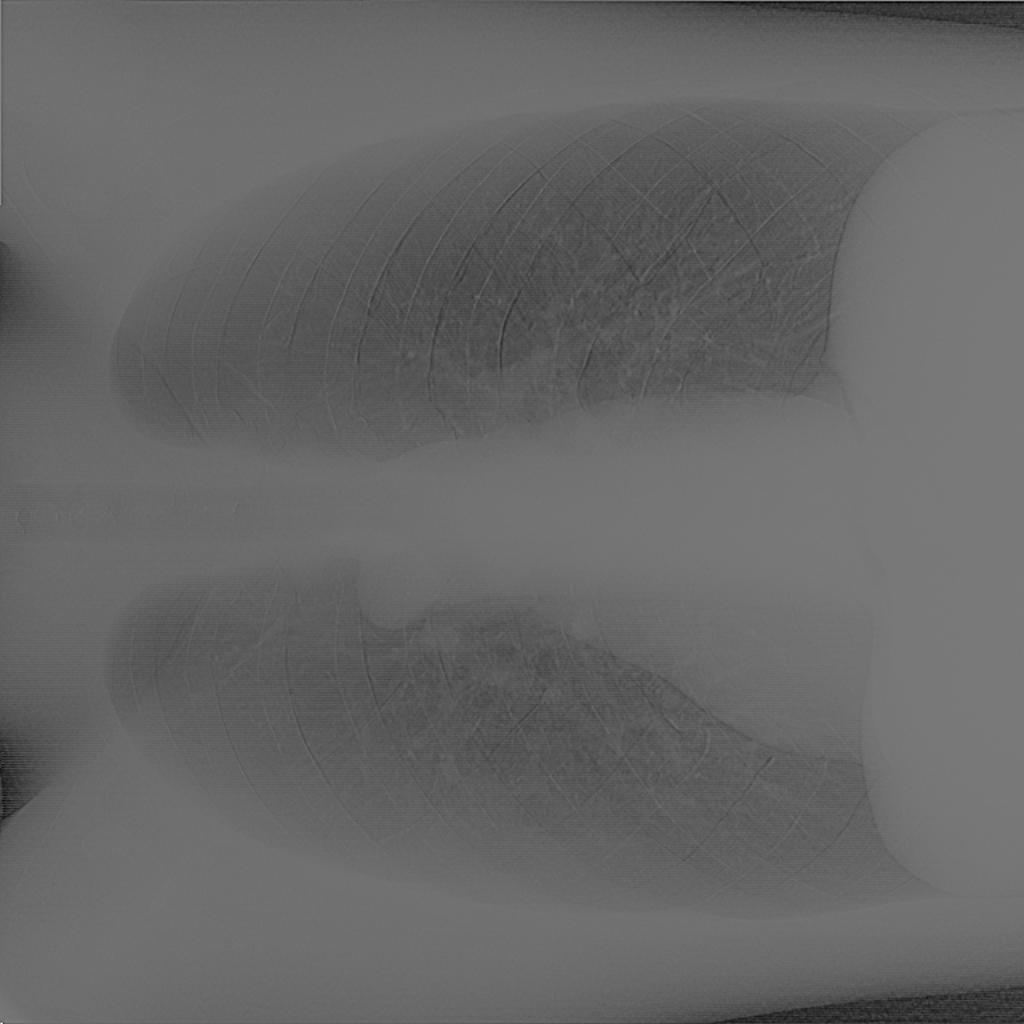

Supplement: S1 File — (ZIP) [file pone.0244745.s001.zip › Figure_1/VM(V).jpg]

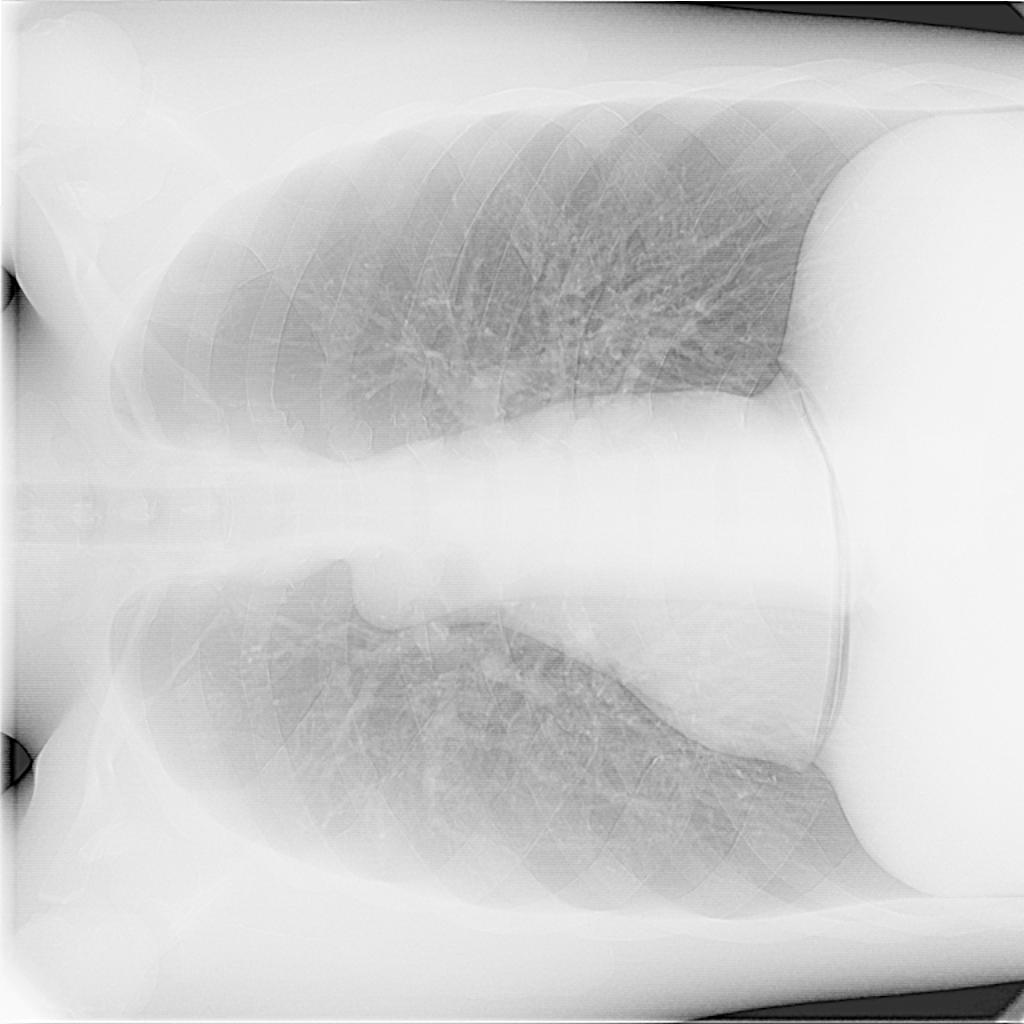

Supplement: S1 File — (ZIP) [file pone.0244745.s001.zip › Figure_3/Interpolated_LR_projection_image.jpg]

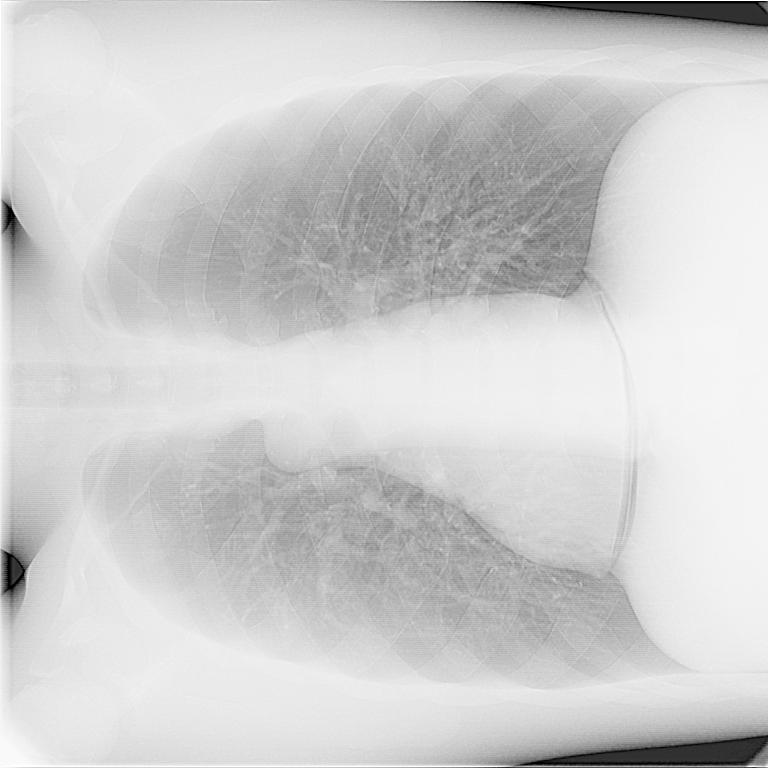

Supplement: S1 File — (ZIP) [file pone.0244745.s001.zip › Figure_3/LR_projection_image.jpg]

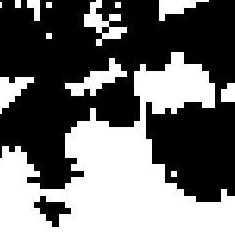

Supplement: S1 File — (ZIP) [file pone.0244745.s001.zip › Figure_5/120_kV_polychromatic_Without_UM_lower.jpg]

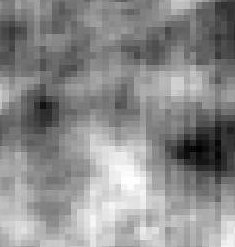

Supplement: S1 File — (ZIP) [file pone.0244745.s001.zip › Figure_5/120_kV_polychromatic_Without_UM_upper.jpg]

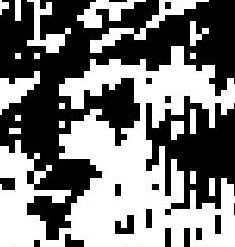

Supplement: S1 File — (ZIP) [file pone.0244745.s001.zip › Figure_5/DE-VM_(60_keV)_With_UM_lower.jpg]

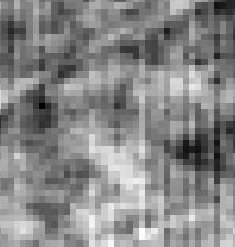

Supplement: S1 File — (ZIP) [file pone.0244745.s001.zip › Figure_5/DE-VM_(60_keV)_With_UM_upper.jpg]

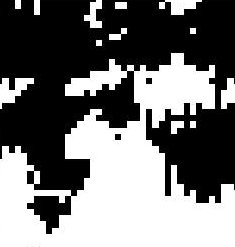

Supplement: S1 File — (ZIP) [file pone.0244745.s001.zip › Figure_5/DE-VM_(60_keV)_Without_UM_lower.jpg]

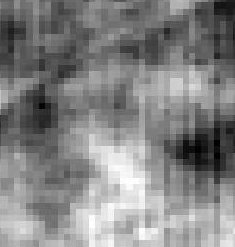

Supplement: S1 File — (ZIP) [file pone.0244745.s001.zip › Figure_5/DE-VM_(60_keV)_Without_UM_upper.jpg]

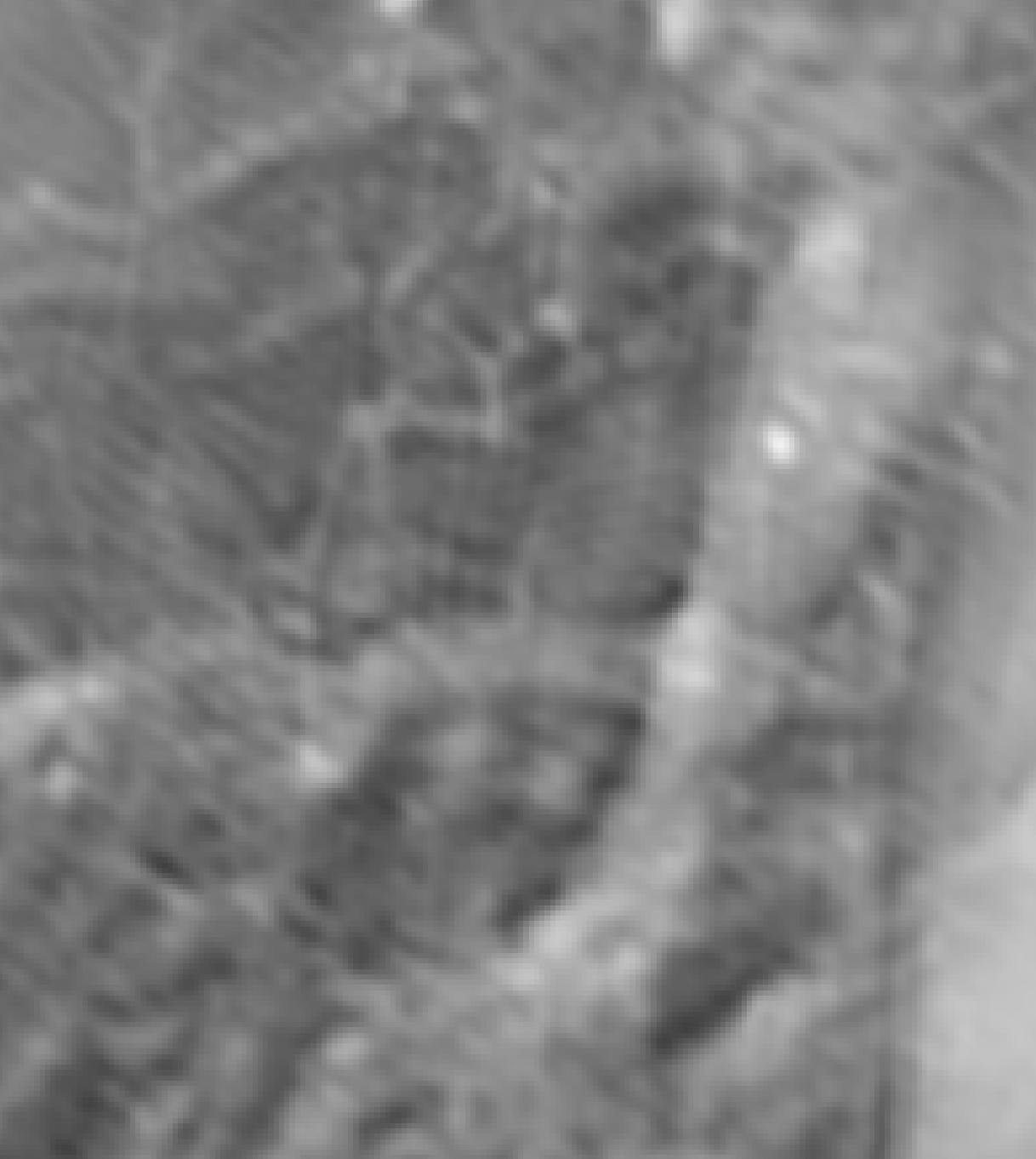

Supplement: S1 File — (ZIP) [file pone.0244745.s001.zip › Figure_6/(a).jpg]

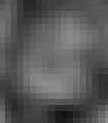

Supplement: S1 File — (ZIP) [file pone.0244745.s001.zip › Figure_6/(b)_&_(c).jpg]

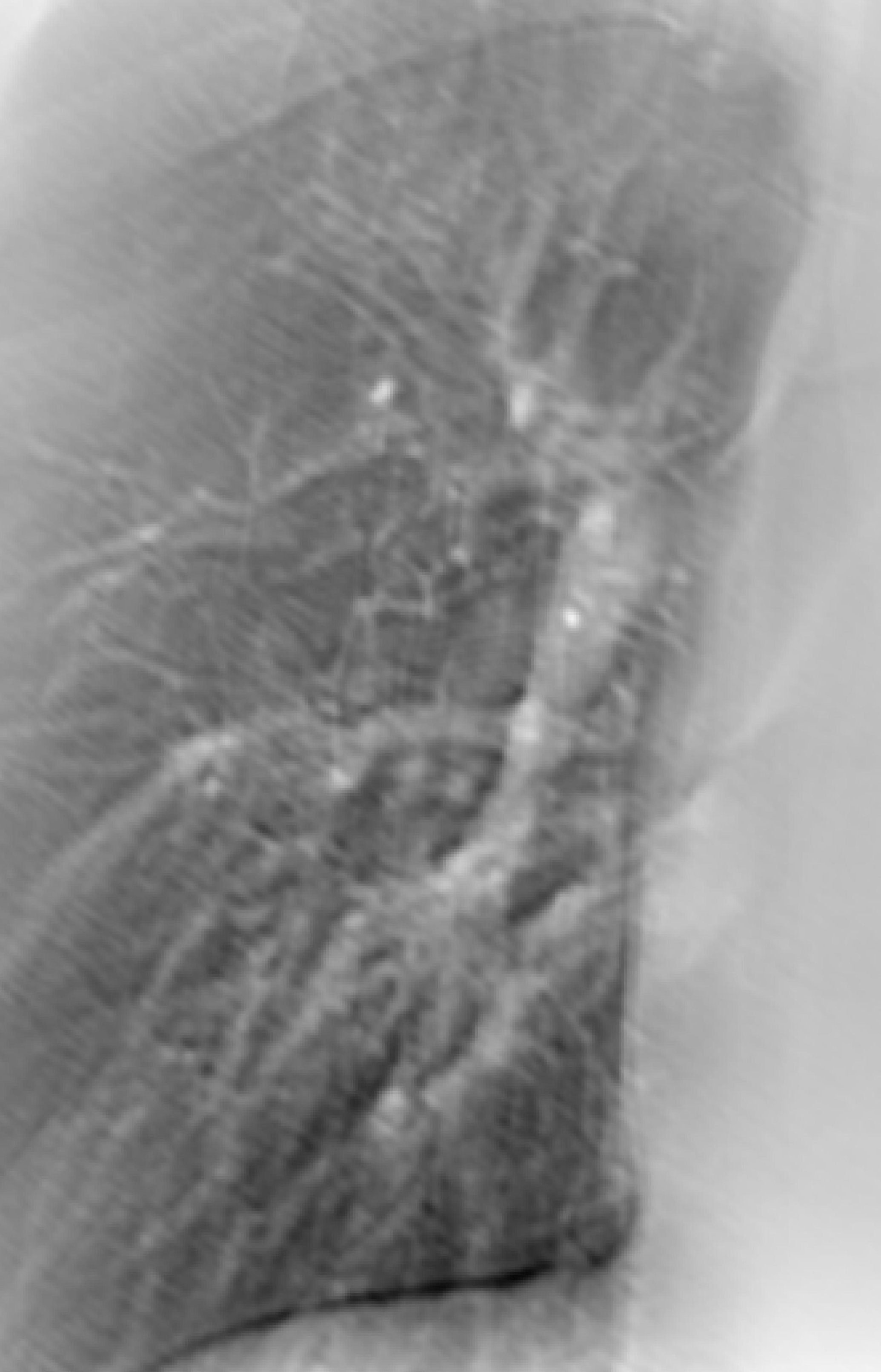

Supplement: S1 File — (ZIP) [file pone.0244745.s001.zip › Figure_6/(In-focus_plane).jpg]

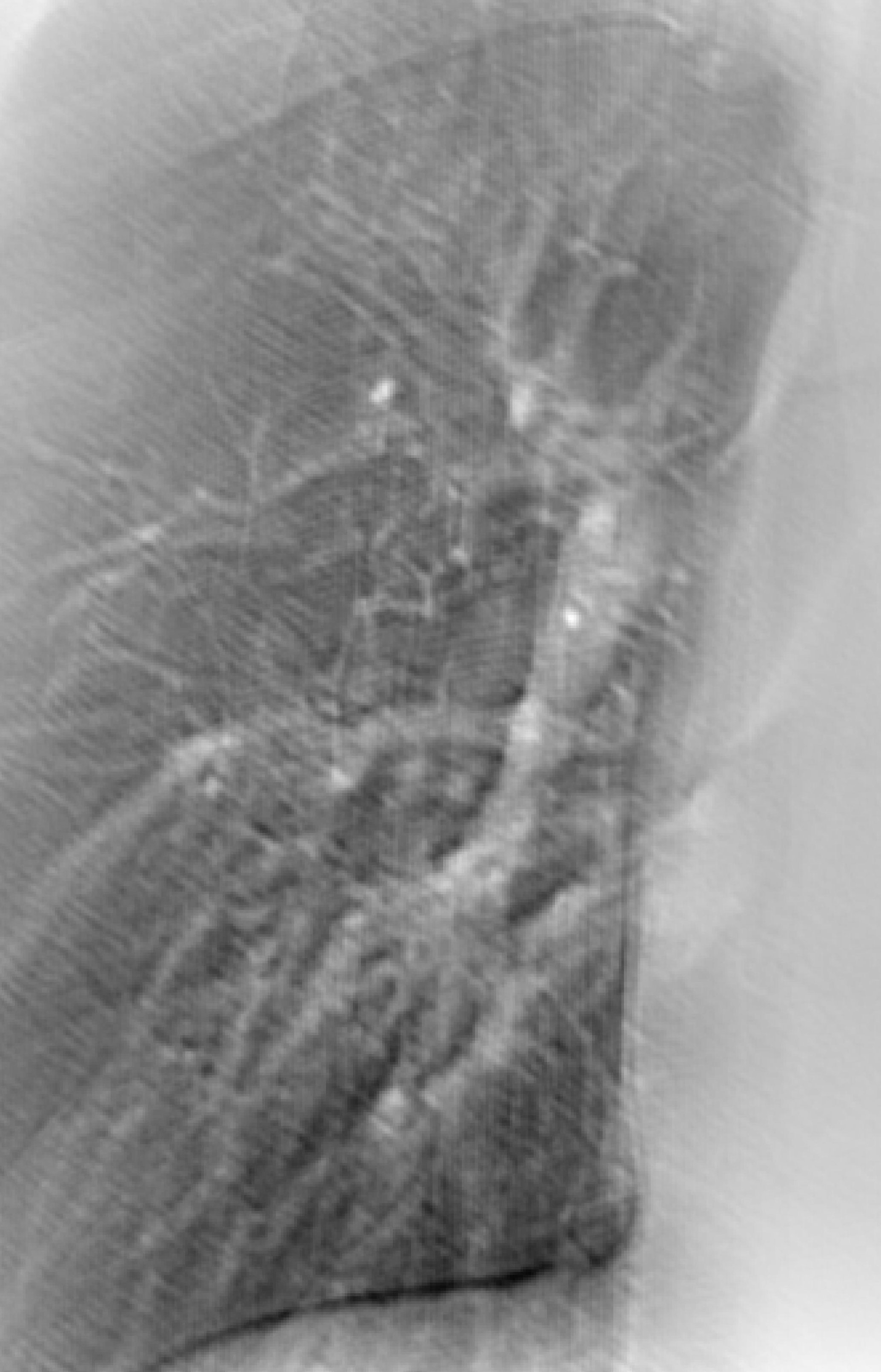

Supplement: S1 File — (ZIP) [file pone.0244745.s001.zip › Figure_9/DE_VM_VDSR_(without_BF).jpg]

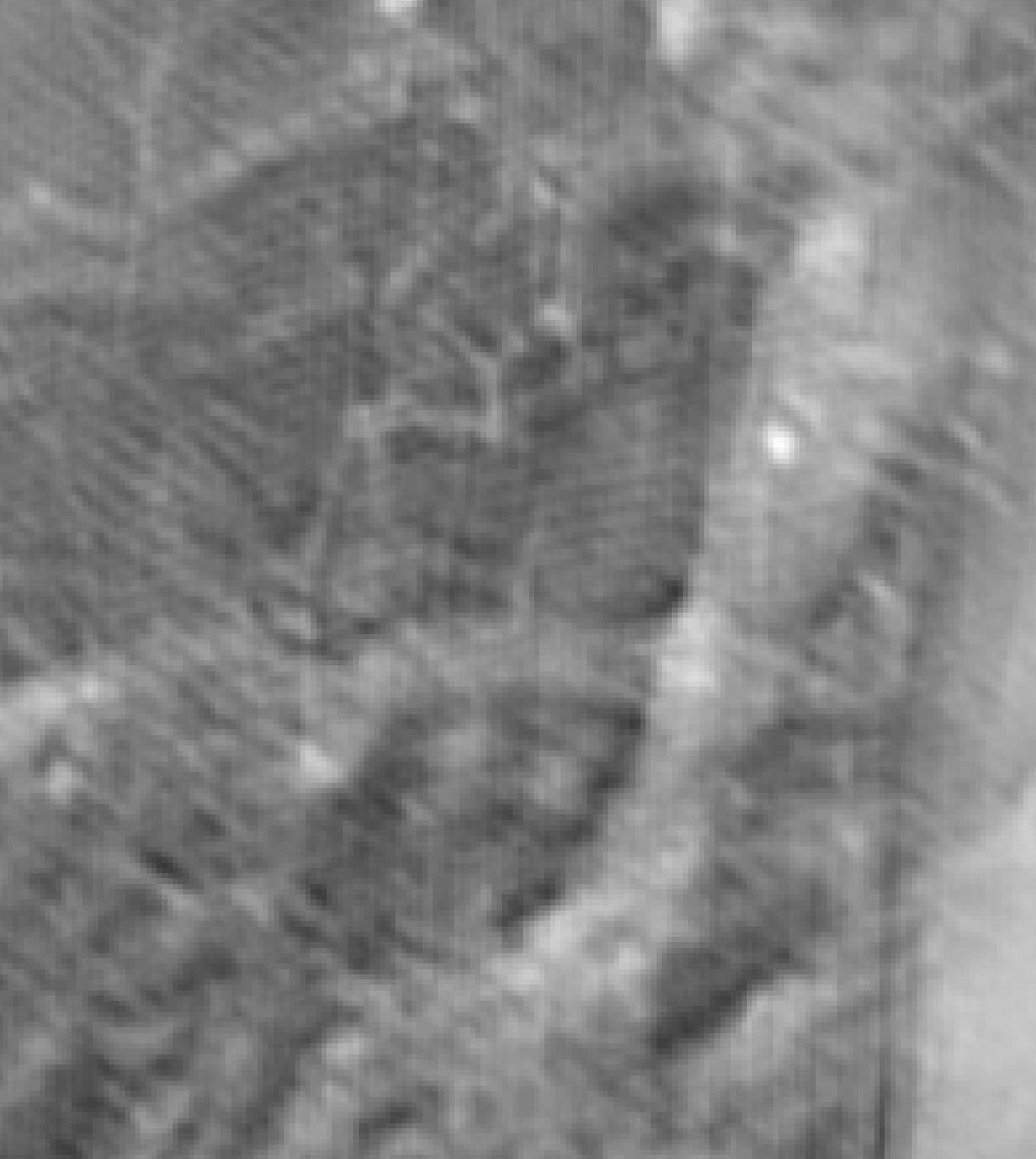

Supplement: S1 File — (ZIP) [file pone.0244745.s001.zip › Figure_9/DE_VM_VDSR_(without_BF)_zoom.jpg]

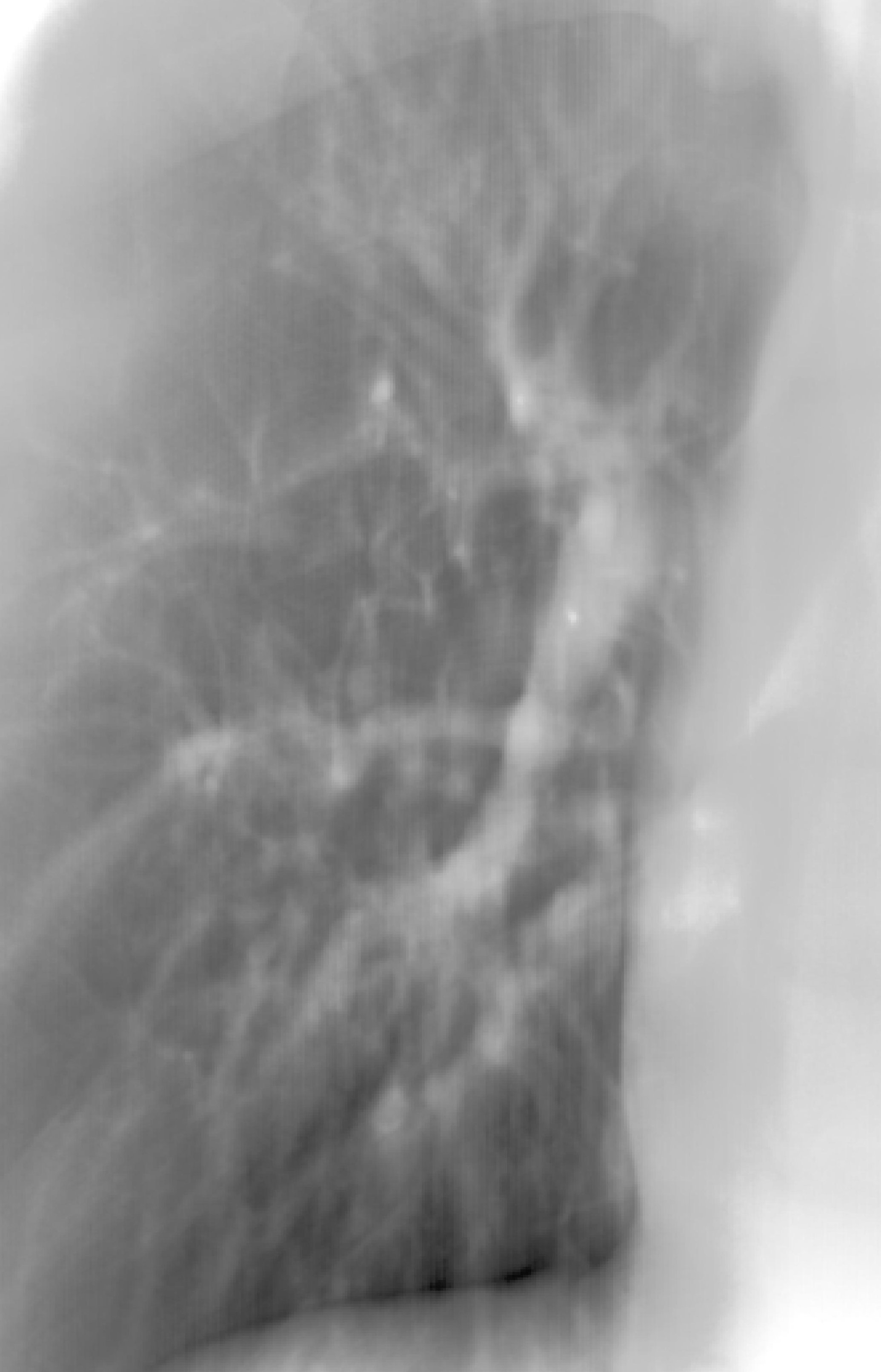

Supplement: S1 File — (ZIP) [file pone.0244745.s001.zip › Figure_9/DE-VM-SART-TV-FISTA.jpg]

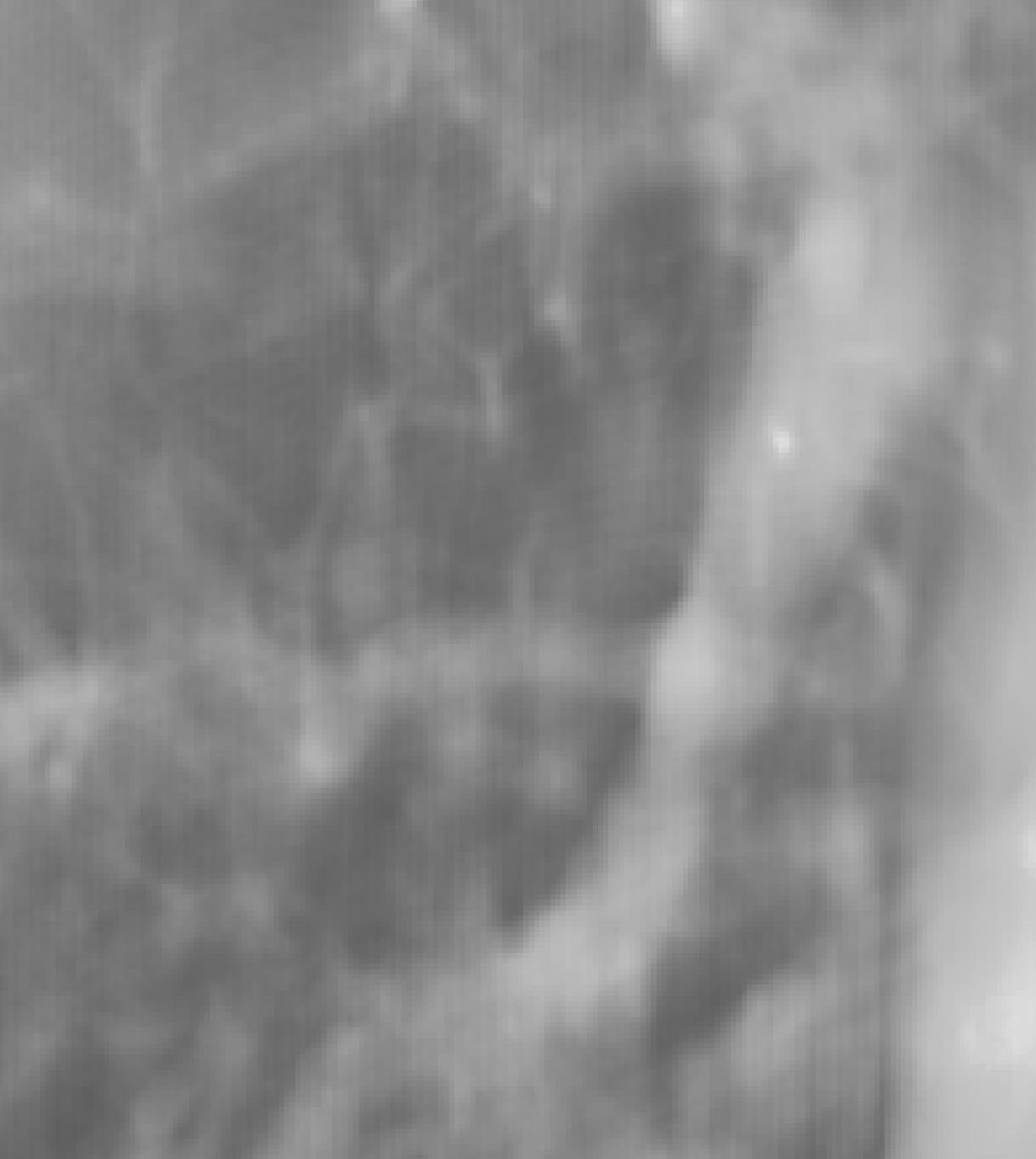

Supplement: S1 File — (ZIP) [file pone.0244745.s001.zip › Figure_9/DE-VM-SART-TV-FISTA_zoom.jpg]

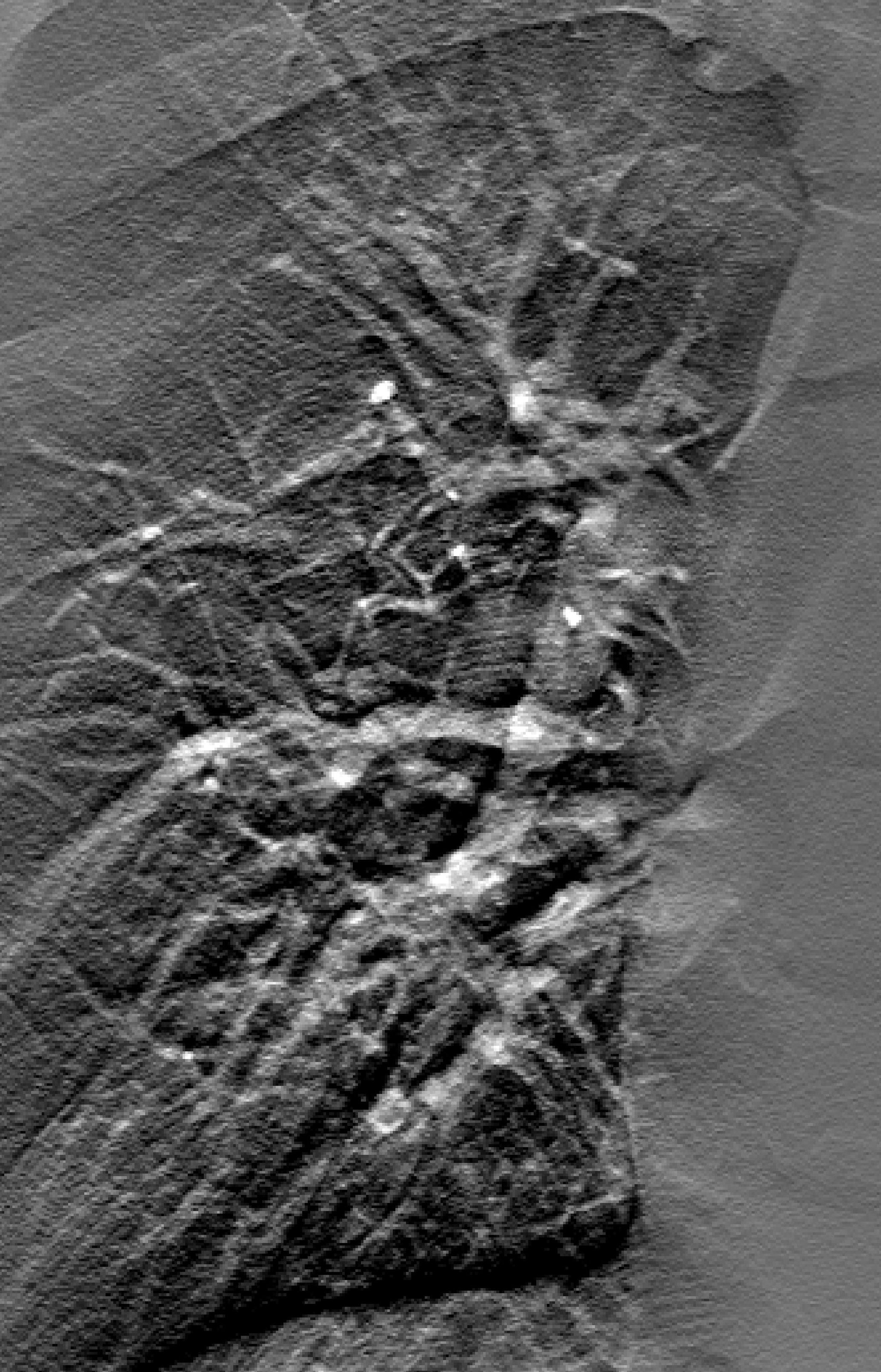

Supplement: S1 File — (ZIP) [file pone.0244745.s001.zip › Figure_9/FBP_ramp_(polychromatic).jpg]

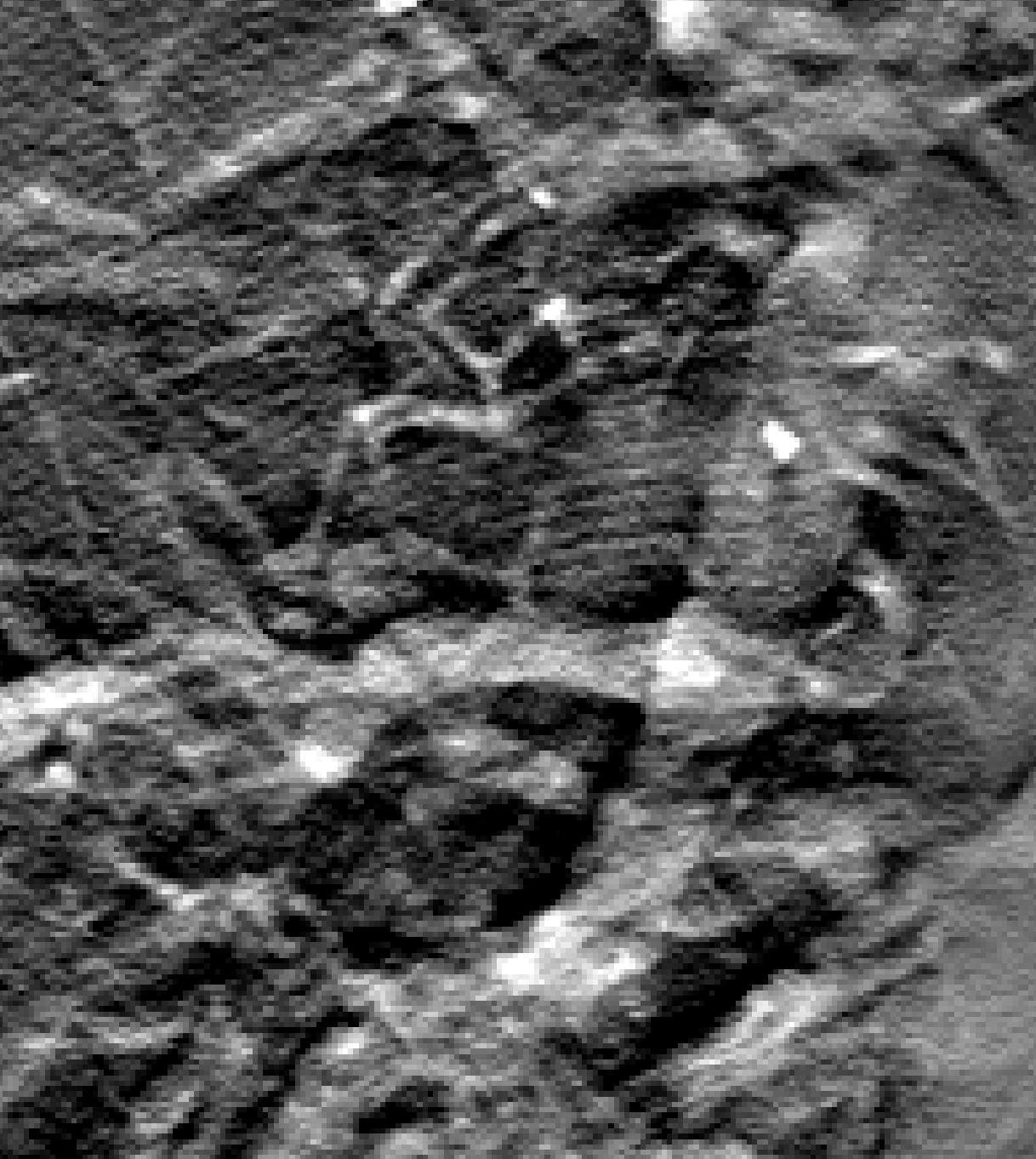

Supplement: S1 File — (ZIP) [file pone.0244745.s001.zip › Figure_9/FBP_ramp_(polychromatic)_zoom.jpg]

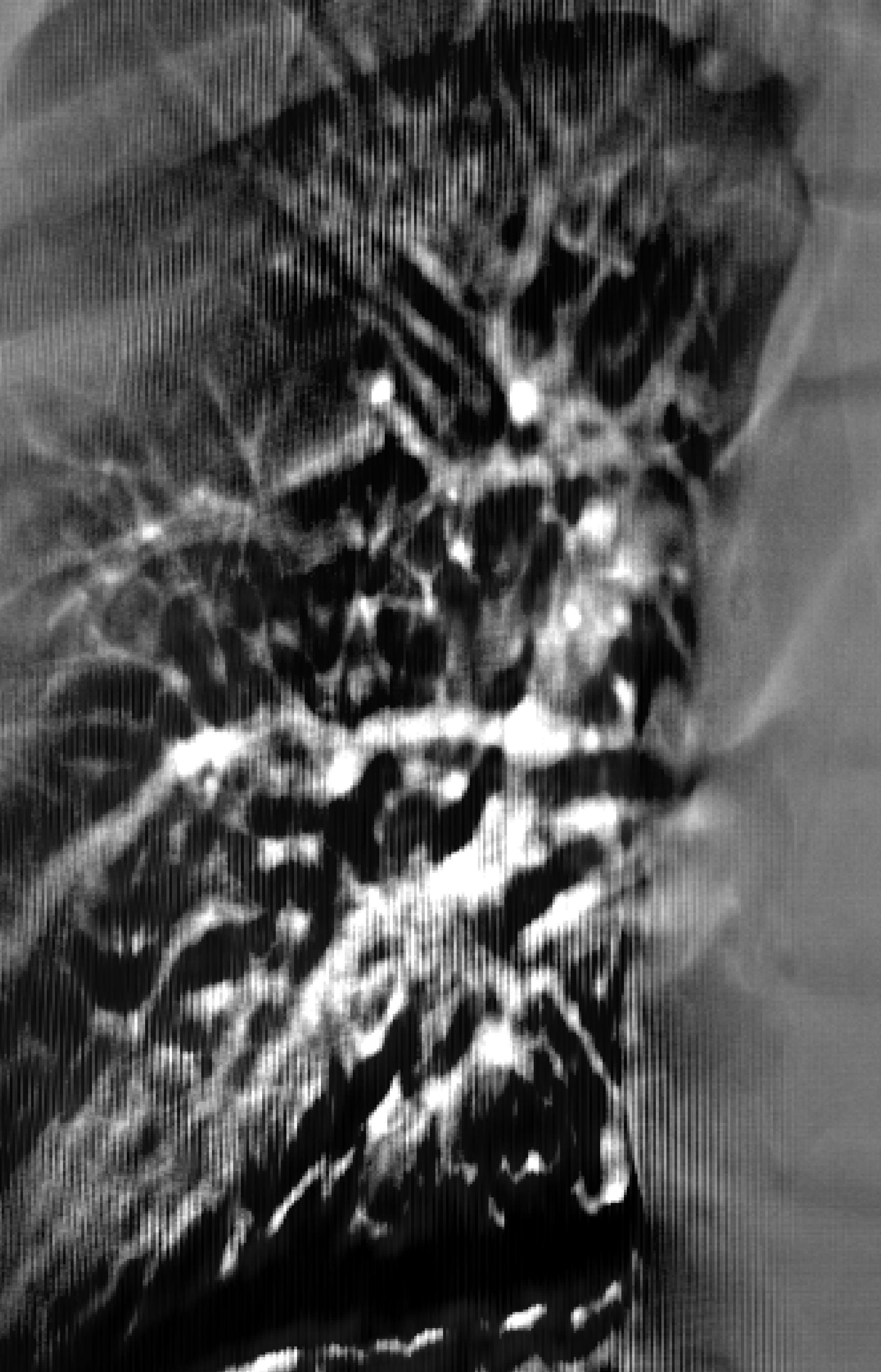

Supplement: S1 File — (ZIP) [file pone.0244745.s001.zip › Figure_9/SART_(polychromatic).jpg]

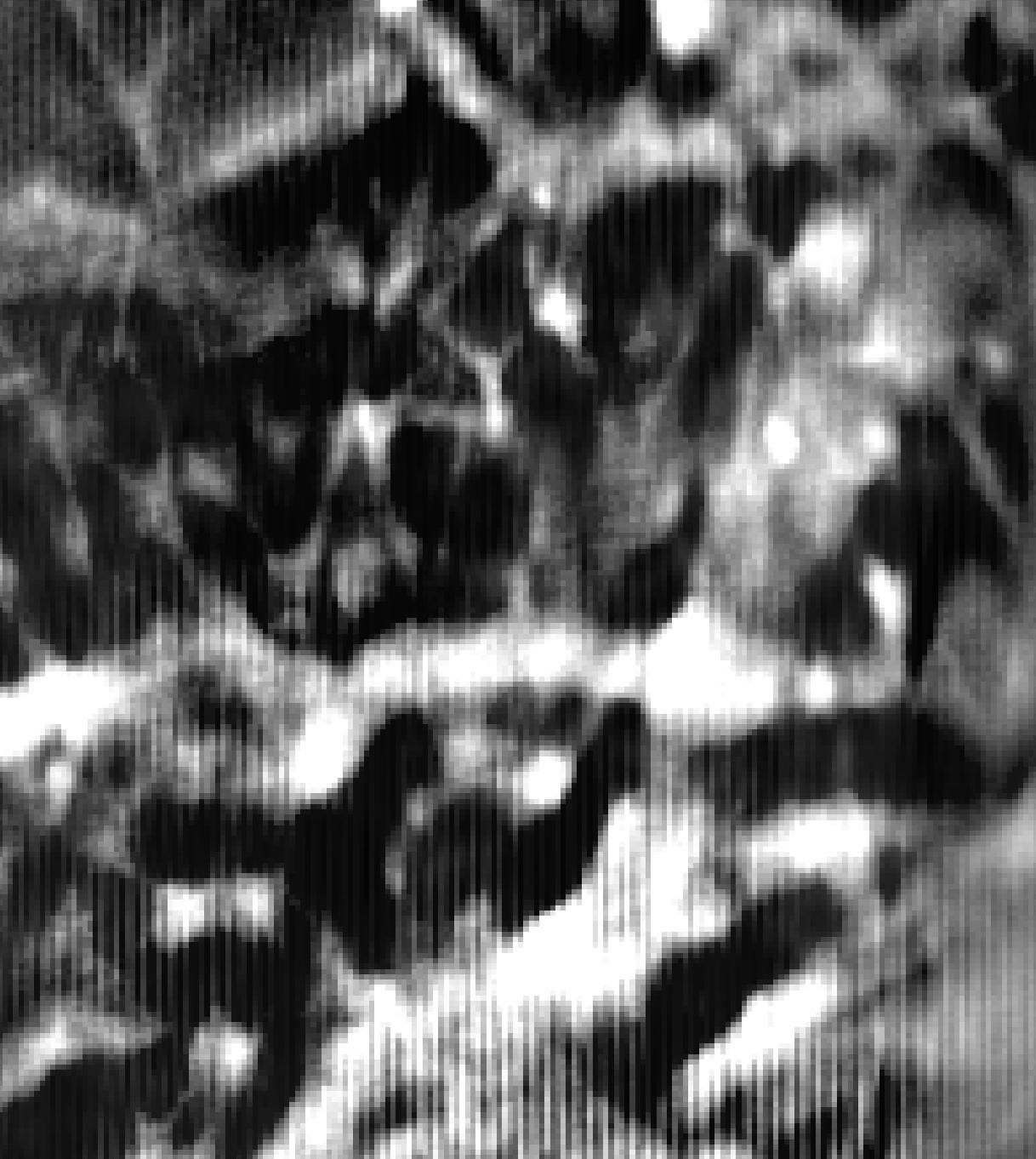

Supplement: S1 File — (ZIP) [file pone.0244745.s001.zip › Figure_9/SART_(polychromatic)_zoom.jpg]

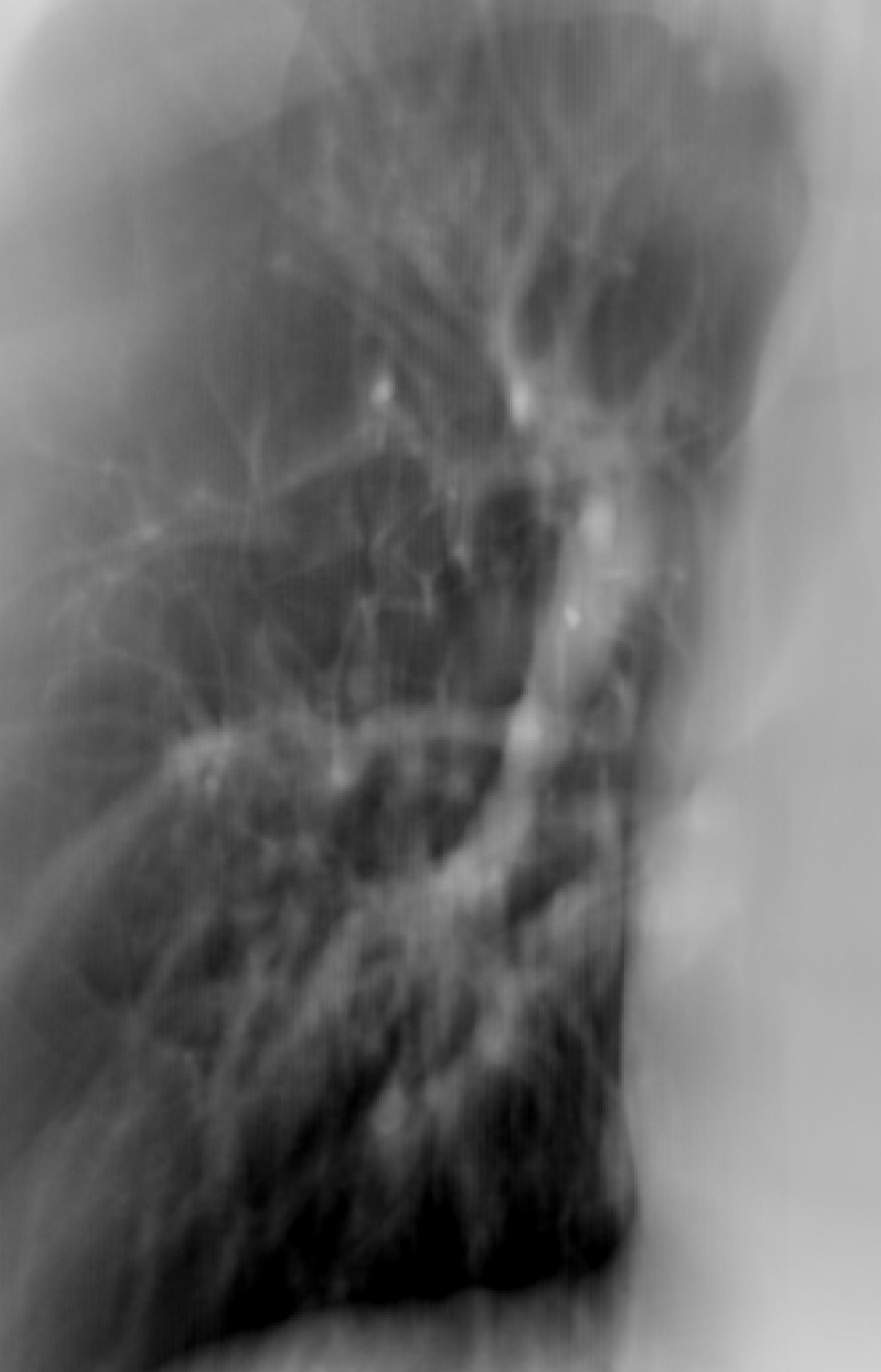

Supplement: S1 File — (ZIP) [file pone.0244745.s001.zip › Figure_9/SART-TV-FISTA_(polychromatic).jpg]

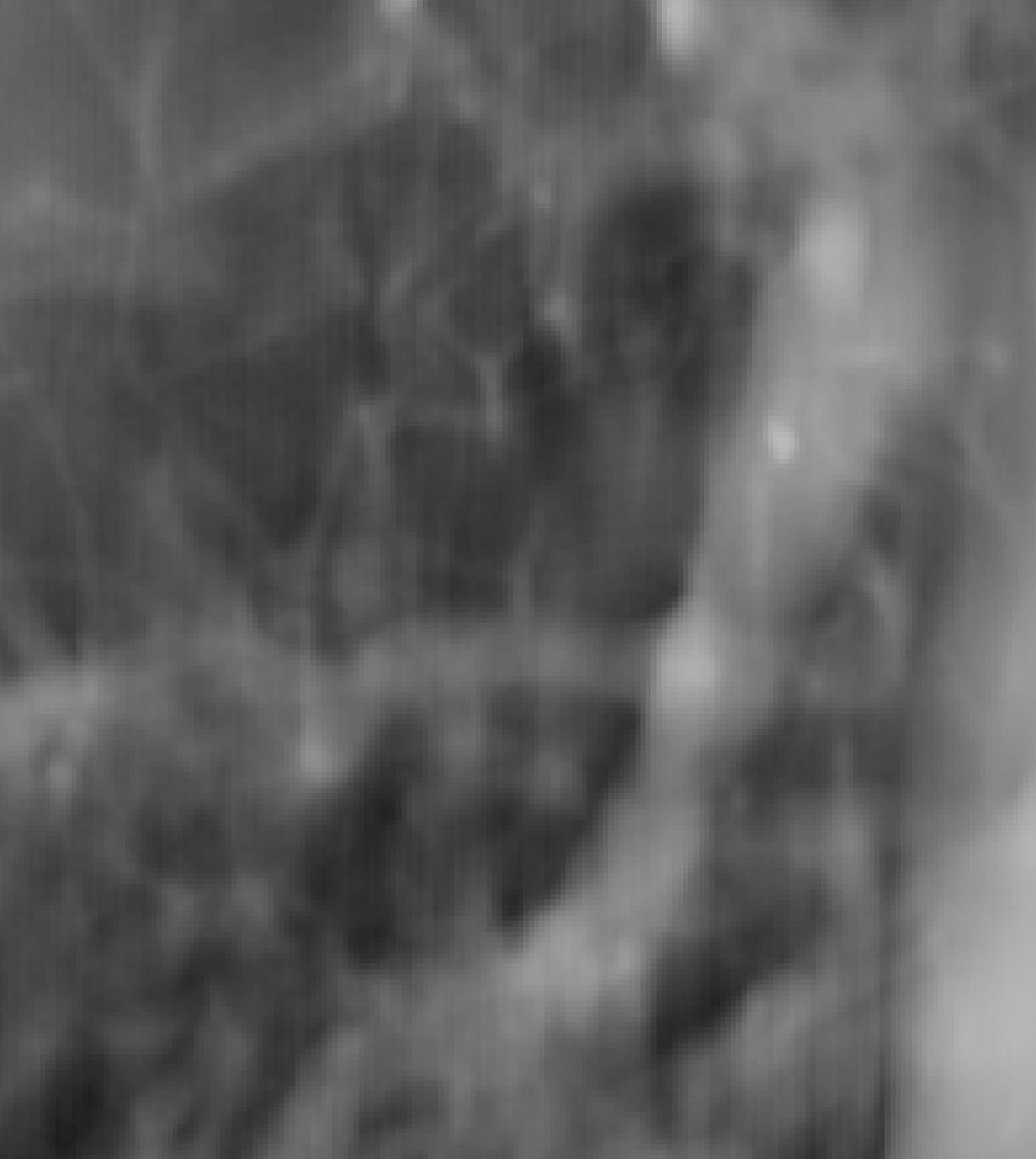

Supplement: S1 File — (ZIP) [file pone.0244745.s001.zip › Figure_9/SART-TV-FISTA_(polychromatic)_zoom.jpg]
