# Supplementary material for: Improved digital chest tomosynthesis image quality by use of a projection-based dual-energy virtual monochromatic convolutional neural network with super resolution
Source: PLoS One. 2020 Dec 31;15(12):e0244745. doi: 10.1371/journal.pone.0244745 (PMC7774945; doi:10.1371/journal.pone.0244745)

Abstract

We developed a novel dual-energy (DE) virtual monochromatic (VM) very-deep super-resolution (VDSR) reconstruction algorithm (DE-VM-VDSR) that uses projection data to improve nodule contrast and resolution and reduce ripple artifacts during chest digital tomosynthesis (DT). For estimating the residual errors from high-resolution and multiscale VM images from projection space, the DE-VM-VDSR algorithm employs a training network (mini-batch stochastic gradient descent algorithm with momentum) that involves subjectively reconstructed and hybrid SR image [simultaneous algebraic reconstruction technique (SART) total variation (TV)-first iterative shrinkage-thresholding algorithm (FISTA); SART-TV-FISTA] and bilateral filtering. DE-DT imaging was accomplished by pulsed X-ray exposures that were rapidly switched between low- and high- tube potential kVp, followed by the comparison of images employing conventional polychromatic filtered back projection (FBP), SART, SART-TV-FISTA, and DE-VM-SART-TV-FISTA algorithms. The improvements in contrast, ripple artifacts, and resolution were compared using a signal-difference-to-noise ratio (SDNR), a Gumbel distribution of the largest variations, and the radial-modulation transfer function (radial-MTF) for a chest phantom with simulated ground-glass opacity (GGO) nodules. The novel DE-VM-VDSR improved overall performance in terms of the SDNR, yielded good images independent of the type of simulated GGO nodules used in the chest phantom, and reduced the ripple artifact. Furthermore, DE-VM-VDSR yielded the least high-frequency ripple artifacts. The novel DE-VM-VDSR yielded superior resolution in comparison to conventional reconstruction algorithms for the radial-MTF analysis, without and with VM processing. Finally, DE-VM-VDSR improved contrast and reduced

high-frequency ripple artifacts.

Introduction

Among the cancer deaths, lung cancer is the main cause, with a significant increase in its incidence globally [1-5]. Although conventional helical computed tomography (CT) is accepted as the gold standard because of its high level of sensitivity for the detection of lung cancer, it has been shown that early-stage lung cancer can be detected by low-dose helical CT, thereby helping to reduce morbidity. In comparison to chest radiography, CT is advantageous as there is less interference by the overlapping anatomical structures which otherwise reduce precision [1]. However, CT has certain drawbacks, in comparison to chest radiography, such as elevated cost and radiation dose. Chest radiography has some advantages as takes much less time for examination and easy availability, but this technique suffers from lower specificity and sensitivity. In chest X-ray, a three-dimensional structure of the chest is projected as a two-dimensional image. Thus, the incomplete detectability of a chest X-ray image is ascribed to the anatomical structures that are overlapping. Even though, chest X-rays are less expensive with lower radiation exposure and easy availability, this technique, due to its low sensitivity, is inadequate for discovering lung nodules because.

Pulmonary nodule detection and characterization in chest imaging is a highly challenging task. It is not uncommon that during examinations of conventional chest radiographs, nearly 30% of such nodules may go undetected by chest radiologists with significant experience [2]. Often, only while reassessing the previous chest images o[illegible] survived patients, nodules are identified retrospectively [3], as it remains difficult to

identify the nodules prospectively. Thus, it can be challenging to assess structures that are not oriented in the CT-axial plane due to partial volume effects (PVE). Because high-resolution CT (HR-CT) reveals the comprehensive organization of local and diffuse substantive aberrations and enables detection of their anatomical distribution, it has greatly improved the ability to evaluate nodules [4, 6, 7]. Conversely, PVE makes lung-field imaging using CT difficult.

Digital tomosynthesis (DT) provides many advantages of digital imaging, including low dose, short duration of examination, PVE-free longitudinal-direction images, and availability without much expense [8-11]. The DT (CDT) requires nearly twice the radiation dose compared to conventional radiography for effective chest examination [12]. In addition to decreasing the radiation dose and expense, CDT offers some of the same advantages provided by CT and can be combined with chest radiography without much difficulty. Like CT, DT reconstructs a longitudinal tomographic image of the subject from a series of projection images obtained from a narrow angle range. DT enhances the ability to detect abnormalities by removing the overlapping anatomical structures and refining the clarity and sharpness of in-plane structures in the images [13]. Dual-energy subtraction (DES) technology has been shown that it can be used in DT chest imaging to enhance detection in comparison to conventional polychromatic imaging [14, 15]. Although DES can produce various images of soft tissue and bone, it increases noise [11, 14]. Yet, due to the intrinsic misalignment of the low- and high-kVp images, digital tomosynthesis (DES-DT) generates a great “rippled” artifact on any visible pulmonary nodules [14, 16]. In CDT, reconstruction from a limited arc angle has been reported to cause ripples making precise diagnosis challenging [14, 16]. Because of this rippled pattern, we anticipate that the pulmonary

nodules may become unnoticed and that if this rippled pattern is overcome, detection of the pulmonary nodules can be improved.

Dual-energy CT (DE-CT) is a possible substitute marker for angiogenesis and regional blood volume, and this has previously been employed for pulmonary nodule characterization, non-invasively [17-19]. DE-CT has been evaluated for lung cancer staging and response assessment in several earlier studies [18]. Presently, the precision of material decomposition is decreased as there are different types of tissues. The relationship between the linear attenuation coefficient and the Hounsfield units of the reconstructed voxel is not specific for any particular material and it changes according to the different properties of the material such as composition, mass density and interaction with photons. However, during DE acquisition, material decomposition can be conducted employing an additional attenuation measurement obtained using different energy spectra. Tissue composition can be differentiated and classified on the basis of information from the alterations in attenuation between the two spectra [20, 21].

Earlier reports indicated the use of DE-CT material decomposition and virtual monochromatic (VM) imaging (projection space approach and image space approach) to enhance nodule detection [17-19, 22]; particularly, enhancements in nodule detection were obtained by the reconstruction of projection data decomposed utilizing the materials in projection space [22, 23]. Even though a DES technique has been described for DT based chest imaging [11, 14, 15], there were no previous reports that described a DE technique with the application of two-material decomposition (bone and soft tissue) to. Low- and high- energy types of weighted blending methods are involved in the two-material decomposition based DE technique. Depending on the chosen blending ratio, either the noise or the contrast may be compromised in this technique. Therefore, a shift

in blending ratio in a given direction will create a blended image with reduced noise, but having lower contrast and information about spatial resolution. Nevertheless, by combining DE technique with three-material decomposition, these inherent problems can be potentially resolved at a high-level.

Deep learning methodologies have been successfully instituted in pattern recognition and processing of images, including denoising [24-26], and super-resolution (SR) [27-29] of images. For example, a convolutional neural network (CNN) has been applied for the enhancement of pulmonary nodule detection in medical imaging by removing residual errors [30-32]. Although these earlier reports demonstrated that a CNN could improve pulmonary nodule detection, no study has used a CNN with CDT. A CNN-based modification (very-deep SR [VDSR]) was presented by Kim et al [33]. A main feature of VDSR includes the reconstruction progress in the learning algorithms. The very-deep architecture combined with procedures of regularization increase image contrast and resolution. The reference and residual images utilized in the training workflow for VDSR are essential for improving nodule contrast and resolution. Physical elements such as beam hardening and X-ray scattering can worsen nodule contrast and resolution. Polychromatic photons containing X-ray beam hardening takes place when it passes through a medium and as the low energy photons are absorbed, resulting in increased mean energy of the X-ray beam. It has been suggested that DE-VM spectral imaging can potentially improve nodule detection [17-19, 22]. We postulate that DE-VM will enhance nodule contrast and resolution thereby improving nodule detection. By conducting residual learning at the projection data level using VDSR processing, an increase in nodule contrast and resolution and decrease in ripple artifacts can be anticipated after reconstruction. To support this proposition, VDSR, which constitutes a

built-in deep feedforward CNN, is employed to produce a SR image from the residual image. However, as VDSR utilizes a method of residual learning, it is also feasible to train the VDSR network architecture to increase nodule contrast and resolution and reduce ripple artifacts. Furthermore, reconstruction employing decomposed projection data from every material (e.g., nodule, soft tissue & vessel, bone) and VDSR-hybrid image processing may augment nodule contrast and resolution and decline of ripple artifacts.

In the present study, we developed a new projection-space-hybrid-reconstruction procedure employing DE-VM with VDSR to increase nodule contrast and resolution and decrease ripple artifacts [DE-VM with VDSR (DE-VM-VDSR)] from CDT.

Materials and Methods

Phantom specifications

The Chest Phantom N1 (Kyoto Kagaku Co., Tokyo, Japan) used in this study consisted of soft tissue and vessels that are constructed of polyurethane resin and artificial bone that is constructed of epoxy resin and $CaCO_3$. Simulated pulmonary nodules used in this study were ground-glass opacity (GGO) type (5 mm in diameter, urethane foam) with a homogenous composition. They were arranged in the right middle lobe region. The simulated nodules placed close to the edges of the lungs or combined with the blood vessels were evaluated. Target contrast (ΔCT) values of the simulated pulmonary nodule (ΔCT = 200 HU) were determined based on the artificial background of the lung parenchyma.

DE-DT system

The DE-DT system (SonialVision Safire II; Shimadzu Co., Kyoto, Japan) consisted of an X-ray tube (anode, that is made up of tungsten with rhenium and molybdenum; real filter: inherent; aluminum [1.1 mm], additional; aluminum [0.9 mm] and copper [0.1 mm]) with a 0.4-mm focal spot and an amorphous selenium (258.05 × 258.05-mm) digital flat-panel detector (detector element, 0.15 × 0.15-mm). The distance between source-to-isocenter was 924 mm while it was 1100 mm between source-to-detector (anti-scatter grid, focused type; grid ratio, 12:1). The selected kV values (low, 60 kV; high, 120 kV) [14] were optimal for testing simulated nodule contrast because they inherently offered optimal tissue decomposition.

1720x1720 detector

During tomography, 37 projection images (1024 × 1024 matrix) were obtained at low- and high-voltage, employing a 40° swing angle and linear system movement, during a single tomographic pass. In DE-DT imaging, exposures with pulsed X-rays were employed with fast interchanges between low and high energies, even though high-voltage is normally deployed for clinical applications [11, 15]. Using the low-voltage X-ray, the projection images were obtained at 280 mA with an exposure time of 27-ms, whereas, the images with high-voltage X-ray were acquired at 416 mA with an exposure time of 2.5-ms. To recreate the tomograms at the needed height, we utilized a 1024 × 1024 matrix with 32 bits (single-precision floating number) per image (pixel size, 0.252 mm/pixel; reconstruction interval, 1 mm).

DE-VM-VDSR

Generation of DE-VM projection images

In this study, we predict that creating VM images using three-material decomposition [34] from DE acquisition can accurately extract nodules, especially those

of GGO type. We performed DE-VM processing at the projection data level to suppress the noise, intending to increase the nodule detection accuracy.

In this study, we used a simple meth[illegible] of projection space (pre-reconstruction) decomposition to evaluate the material fractions F_n of the artificial bone ($CaCO_3$ + $C_{15}H_{16}O_2$, F_c, local density; 1.3098 g/cm^3), soft tissue ($O_4N_2H_2$, F_p, local density; 1.0600 g/cm^3), and simulated GGO nodule ($C_3H_8N_2O$, F_u, local density; 0.3500 g/cm^3) in the phantom.

By using the density corresponding to each of the three basic materials containing areas, the linear attenuation coefficient $\mu(E)$ can be calculated for any photon energy E. The theoretical linear attenuation coefficient curve depicted in Fig 1 was determined employing each material's local and area densities (g/cm^2). These are produced using the XCOM program designed by Berger and Hubbell [35]. Finally, for the projection space, we employed decomposition approach for producing decomposition images for materials, such as urethane foam, polyurethane, and artificial bone. The material fractions F_n were derived from the inverse of this matrix. [illegible]nverse of this matrix [the "*inv*" function from MATLAB (Mathworks; Natick, MA, USA) was used; this function limits the possible fraction to [0,1] while imposing a sum of 1] was utilized to acquire material fractions. Thus, from the processing pipeline, three-material fractions that are related to artificial bone, soft tissue, and simulated GGO nodules, arise. DE-VM processing is obtained using the equation (1):

$$V = F_u \cdot \left(\frac{\mu}{\rho}\right)_u (E) + F_p \cdot \left(\frac{\mu}{\rho}\right)_p (E) + F_c \cdot \left(\frac{\mu}{\rho}\right)_c (E) \qquad (1)$$

$$F_u + F_p + F_c = 1.0$$

Where V is the DE-VM projection image, and $(\mu/\rho)_u(E)$, $(\mu/\rho)_p(E)$, and $(\mu/\rho)_c(E)$ are each material's corresponding mass attenuation coefficients. We evaluated the optimal DE-VM-energy (keV) in the "Optimization parameters" section below.

Fig.1

Linear attenuation coefficients for each energy. The linear attenuation coefficients of a simulated ground-glass opacity (GGO) nodule, soft tissue, and artificial bone with respect to the photon energy.

VDSR

In medical imaging, reports have shown improvements in resolution using CNN. In DT, enhancing the clarity and sharpness of the DE-VM-projection image is a way to augment nodule detection. Therefore, we recommend to apply CNN-based deep learning processing to DE-VM-projection images to improve resolution (so-called SR). Enhancing image resolution by SR is useful to more precise detection of nodules.

SR is a method for generating images with high-resolution (HR) from low-resolution (LR) images with the goal of recovering one HR image from one LR image. Single-image SR is difficult as it is not possible to retrieve high-frequency image content from the LR image. In the absence of high-frequency information, the HR image quality is limited. VDSR is a CNN architecture developed to conduct single-image SR [33]. The VDSR network learns the mapping between low- and high-resolution images. LR and HR images differ mainly in high-frequency details, even though they have similar image content, which makes this mapping possible.

VDSR uses a residual learning strategy, i.e., this network trains to assess a residual image. In the context of SR, a residual image is the difference between an HR image and an LR image that has been upscaled using bicubic interpolation to match the size of the HR image. A residual image comprises information regarding the high-frequency details of an image.

VDSR is a state-of-the-art SR deep learning approach for enlarging an image [33]. Digitally enlarging (zoom in) a poor image leads to image blurring and poor image quality because the conventional interpolation or enlargement algorithm is insufficient. However, VDSR can obtain a HR image with high quality from a LR image.

Training dataset

Thirty-seven reference images (comprising of upscaled images and the corresponding residual images) related to the revised input image pairs were selected randomly as the training set from the generated VM projection data (total training data set: 74). Different scale factors (2, 3, or 4) [33] were used to create sample LR images; subsequently, the images were resized to the original size (1024 × 1024 matrix) using bicubic interpolation. The difference between the original and resized images was determined. The network inputs were LR images, upscaled employing bicubic interpolation.

Architecture

There are 20 weight layers in the network, and these layers are all identical with the exception of the first and last. A majority of the network consists of 18 conventional layers, each layer with 64 3 × 3 filters, followed by a rectified linear unit (ReLu) [36]. The desired residual image is reconstructed by the final layer, with a 3 × 3 × 64 filter [33].

This final residual image can then be merged with the LR input to generate the resulting SR output (Fig 2).

Fig. 2

Network architectures. Convolutional neural network for chest digital tomosynthesis super resolution.

Residual learning

G denotes an HR image and H denotes an interpolated LR image. In this study, we propose to apply unsharp-masking (UM) processing to the image (original VM image: V) before processing the interpolated LR image (H). To maintain a balance between high-frequency noise and edge preservation, the parameter (standard deviation) of UM processing was set to 2.5. We anticipate that applying UM processing to the original image will further improve the sensitivity of detecting lesions like a simulated GGO nodule. Interpolated LR images are generated using the following equation;

$$H = D_{\downarrow}\left[k \cdot \left(V - V_{g\_lowpass}\right) + V\right] \quad (2)$$

$D_{\downarrow}$: Downscaling interpolation operator

k : Scaling contrast

$V_{g\_lowpass}$: Gaussian low pass filtered image

In a learning-based context, the training dataset $\{G_{\tau}, H_{\tau}\}_{\tau=1}^{\omega}$ is used to learn the mapping from the LR images H to the HR images G, resulting in the following equation;

$$\hat{G} = \arg\min_{G} \|H - ZG\|^2 \quad (3)$$

Z : Observation matrix $[Z \in \Re^{\Gamma \times \Omega}, (\Gamma = \Omega)]$

Given a set of HR images G_τ $(G \in \Re^{\Gamma})$ and their corresponding LR images H_τ $(H \in \Re^{\Omega})$ with ω samples, the restoration operator f can be estimated as follows;

$$\hat{f} = \arg\min_f \sum_{\tau=1}^{\omega} \frac{1}{2} \left\| G_\tau - f(S^{\uparrow} H_\tau; \delta) \right\|^2 \quad (4)$$

$S^{\uparrow}$: Upscaling interpolation operator

δ : Trainable parameter

The network is learning the residual errors between the output (HR image) and input (LR image) instead of learning the HR output.

The VDSR training workflow can be understood by using the mini-batch stochastic gradient descent algorithm with momentum (SGDM) method [37]. The restoration function f to learn the trainable parameters δ [SGDM with the weight decay (L_2 penalty) of 0.0001, momentum of 0.9, initial learning rate of 0.1, and hyper-parameters mini-batch size and epochs] in the VDSR [33]. The hyper-parameters of δ that affect contrast are mini-batch size and epochs [33]. We determined the optimal parameters and applied them in the "Optimization parameters" section below.

f is a mapping from the interpolated LR image to the HR image. VDSR is used to estimate high-frequency components from the LR to the HR and to estimate a mapping from the LR to the missing high-frequency components, also called residual r which is between HR and LR; $r = G - \left(S^{\uparrow} H\right)$. This method can be modeled by a skip connection in the network. Residual-based modeling assumes that r is a function $S^{\uparrow} H$. The final

SR is then depicted as below;

$$G = S^{\uparrow}H + f\left(S^{\uparrow}H\right) \tag{5}$$

Where, f can be determined using the following equation;

$$\hat{f} = \arg\min_{f} \sum_{\tau=1}^{\omega} \left\| \left(G_{\tau} - \left(S^{\uparrow}H\right)_{\tau}\right) - f\left(\left(S^{\uparrow}H\right)_{\tau}\right) \right\|^{2} \tag{6}$$

Multiscale

After the VDSR network learns to assess the residual image, it can recreate HR images by combining the assessed residual image with the upscaled LR image. ~~As the scale factor is increased, single-image SR turns out to be more ill-posed as the LR loses more details about the content of high-frequency image; however, VDSR provides solution to this problem~~. To produce the corresponding LR images, the HR images were down-scaled. In this study, scale factors of 2, 3 or 4 were used for training and the highest contrast was measured for each scale factor during testing [33]. After downscaling, they were then upscaled back to their previous resolution, resulting in a LR image dataset that is suitably distorted. From each of these images, 64 41 × 41 image patches were retrieved, some of which were then rotated randomly by 90° [33]. Data augmentation (random rotation by 90 degrees and random reflections in the horizontal direction) was used during training to alter the training data, essentially to increase the volume of available training data (randomly select a scale factor as a form of data augmentation). The training model extracts randomized patches from the upscaled and residual images. This type of data enhancement is often employed for image-to-image regression problems, where multiple network architectures can be trained employing very small input image sizes. Thus a large number of patches can be retrieved from each full-sized image in the original training set,

volume/number

significantly enhancing the training set size. This extraction and rotation of patches facilitates the network to train and ensures a suitable diversity in its training data.

FISTA

Although filtered back projection (FBP) [13] has excellent detection sensitivity in the high-frequency region, it is a concern that noise and ripple artifacts are enhanced. In contrast, a simultaneous algebraic reconstruction technique (SART) [38] is expected to be effective at suppressing noise in iterative reconstruction (IR), and its usefulness has been reported in various fields [39-42]. The usefulness of combining first iterative shrinkage-thresholding algorithm (FISTA) and IR-total variation (TV) has been recently reported [43, 44]. By combining FISTA, IR-TV, and deep learning, it is thought that an integrated and improved image can be generated. We adopted a combined reconfiguration, SART-TV-FISTA, for this study.

SART-TV-FISTA algorithm consists of three steps: 1) SART update, 2) TV-minimization, 3) FISTA acceleration technique. The reconstruction process of SART is defined by;

$$s_j^{(k+1)} = s_j^k + \lambda \cdot \frac{\sum_{g_i \in G_\theta} \frac{g_i - \sum_{n=1}^{N} b_{in} \int_n^k s}{\sum_{n=1}^{N} b_{in}} \cdot b_{ij}}{\sum_{g_i \in G_\theta} b_{ij}} \quad (7)$$

Where s_j refers to a voxel in the object function s, g represents a single pixel in the DE-VM-SR projection data G, θ is the projection angle of view, b_{ij} is an element in the matrix B, k is the iteration index and λ is relaxation factor. TV-minimization is a problem of optimization that was motivated by the compressed sensing (CS) theory [45]. This technique has been applied to tomography [46], and is expressed as

$$\vec{s}^* = \arg\min \|s\|_{TV} \tag{8}$$

$$\left| B \cdot \vec{s}^* - hr \right| < \delta \tag{9}$$

$$\|s\|_{TV} = \sum_{i,j,k} \sqrt{\left(s_{i,j,k} - s_{i-1,j,k}\right)^2 + \left(s_{i,j,k} - s_{i,j-1,k}\right)^2 + \left(s_{i,j,k} - s_{i,j,k-1}\right)^2} \tag{10}$$

Where $\vec{s}^*$ is the volume image to be recreated, δ is a parameter that relates to the level of data inconsistency tolerance and $\|s\|_{TV}$ is the $l_1 - norm$ of the image gradient magnitude which is used as the cost function. $s_{i,j,k}$ is the value of the voxel with index (i, j, k). Standard techniques like conjugate gradients or steepest descent were employed to solve Eqn (8). The steepest gradient method, which is based on an iterative scheme, can be used to minimize the TV objective function. In this method the image is updated at each iteration n $(n = 1 \ to \ 20)$ as follows:

$$s^{(n+1)} = s^{(n)} - \beta \cdot d_p \cdot \frac{ds^n}{\left|ds^n\right|} \tag{11}$$

$$d_p = \left|s - s_0\right|$$
$$ds^n = \nabla s \|s\|_{TV}$$

The value of d_p is computed initially and depends on the values of s_0 and s corresponding to the image values before and after the SART update step, respectively. ∇ represents the gradient operator and β is a parameter to control the effect of regularization. The regularization parameter β was set to 1e-7 to maximize the simulated nodule contrast. The FISTA acceleration step is used for convergence and is denoted by

$$s^{(m+1)} = s^m + \left(\frac{t^m - 1}{t^{(m+1)}}\right)\left(s^m - s^{(m-1)}\right) \tag{12}$$

Where the parameter t is updated each iteration as follows

$$t^{(m+1)} = \frac{1 + \sqrt{1 + 4\left(t^m\right)^2}}{2} \tag{13}$$

$t = 1$ is the initial given value and m is the index of the global iteration step.

Bilateral filtering

The bilateral filter performs smoothing while preserving edges such as contours, and is applied to enhance image quality by lowering noise in medical images [40]. The purpose of using a bilateral filter in this algorithm is to suppress and adjust the increase in high-frequency components (especially, noise) after reconstruction. The bilateral filter of the identified in-focus plane image is defined by the equation below.

$$BF(i, j) = \frac{\sum_{\gamma=-\eta}^{\eta}\sum_{\varepsilon=-\eta}^{\eta} s(i+\varepsilon, j+\gamma)\exp\left(-\frac{\varepsilon^2+\gamma^2}{2{\sigma_d}^2}\right)\exp\left(-\frac{(s(i,j)-s(i+\varepsilon, j+\gamma))^2}{2{\sigma_R}^2}\right)}{\sum_{\gamma=-\eta}^{\eta}\sum_{\varepsilon=-\eta}^{\eta} \exp\left(-\frac{\varepsilon^2+\gamma^2}{2{\sigma_d}^2}\right)\exp\left(-\frac{(s(i,j)-s(i+\varepsilon, j+\gamma))^2}{2{\sigma_R}^2}\right)} \tag{14}$$

Where, η is a set of neighborhood points around the pixel, σ_d is the standard deviation of domain filter, σ_R is the standard deviation of the range filter, BF is applied in VDSR with DE-VM, SART-TV-FISTA, and bilateral filtering of final DE-VM-VDSR image. The parameters are a set of η [$\eta = 2\cdot(2\cdot\sigma_d)+1$: σ_d =1; 5, σ_d =2; 9, σ_d =3; 13], and σ_R ($\sigma_R = 0.01\cdot[0\ ,\ 1.0]^2_{img\_scale\_diff}$, $[\ ,\ ]_{img\_scale\_diff}$; scale range of image, $\sigma_R = 0.01$). The parameter that must be considered in the effect of the bilateral filter on contrast is σ_d. Fig 3 shows a flowchart of the DE-VM-VDSR algorithm. Furthermore, Fig 4 explains the relationship between VDSR, SART-TV-FISTA, and bilateral filtering, which are the

cores of the reconstruction process. We assessed the optimal parameter σ_d , and applied the parameters in the "Optimization parameters" section given below.

Fig. 3

Flowchart of the dual-energy (DE) virtual monochromatic (VM) with very-deep super-resolution (VDSR) reconstruction algorithm (DE-VM-VDSR). The novel DE-VM-VDSR algorithm was implemented in projection space data with the aim of increasing nodule contrast and resolution while reducing ripple artifacts during digital chest tomosynthesis (DT). The DE-VM-VDSR algorithm is based on a training network (mini-batch stochastic gradient descent algorithm with momentum) to estimate the residual image from high-resolution and multiscale VM images in projection space, involving hybrid and subjectively reconstructed SR image [simultaneous algebraic reconstruction technique (SART) first iterative shrinkage-thresholding algorithm (FISTA)-total variation (TV); SART-TV-FISTA] and bilateral filtering.

Fig. 4

Overview and interrelationship of each algorithm. The flowchart shows the interrelationship of very-deep super-resolution (VDSR), the simultaneous algebraic reconstruction technique total variation first iterative shrinkage-thresholding algorithm (SART-TV-FISTA), and bilateral filtering which are the core of the dual-energy (DE) virtual monochromatic (VM) with very-deep super-resolution (VDSR) reconstruction algorithm (DE-VM-VDSR).

Evaluation

Optimization parameters of VM-energy, mini-batch size, epochs, number of iterations (k ,m) for iterative reconstruction, and standard deviation of domain filter (σ_d)

The FBP (kernel: Ramachandran-Laksminarayanan) and SART (polychromatic 120 kV acquisition) were used as in previous chest DT studies [41]. It is necessary to optimize the number of iterations since the characteristics of the IR image depend on the number of iterations. First, optimization of iteration number is performed in polychromatic SART and SART-TV-FISTA using root-mean square error (RMSE). The RMSE of the identified in-focus plane image is given as shown below:

$$RMSE = \sqrt{\frac{1}{\tau\varsigma}\sum_{i=0}^{\tau-1}\sum_{j=0}^{\varsigma-1}\left[K_{ref}(i,j) - V_{obj}(i,j)\right]^{2}} \qquad (15)$$

Where, $K_{ref}(i,j)$ is the (i,j) th entry of a current iteration image and $V_{obj}(i,j)$ is the (i,j) th entry of a previous iteration image of each algorithm.

Then, the optimal iteration number of the polychromatic (120 kV) IR image was applied to monochromatic SART-TV-FISTA (DE-VM-SART-TV-FISTA) and DE-VM-VDSR. The evaluation on the optimization of DE-VM-energy, mini-batch size, epochs, and standard deviation of the domain filter was performed using signal-difference-to-noise ratio (SDNR) on in-focus plane image. The SDNR of the identified in-focus plane image is given as indicated below:

$$SDNR = \frac{\left|\Lambda_{GGO} - \Lambda_{BG}\right|}{\sigma_{GGO}} \qquad (16)$$

where, Λ_{GGO} and Λ_{BG} were mean pixel intensities inside the simulated GGO nodule and background (BG) fields, respectively, and σ_{BG} was the standard deviation of multiple ROIs (radius: 4 pixel, area: 50 pixel) in the BG field. Multiple circular ROIs inside the lung field represented the background signal [Fig 5 (a)]. Reconstruction was achieved employing DT system-derived real projection data. MATLAB (Mathworks) was utilized for the reconstruction and processing of images. Optimization was evaluated

using the RMSE and SDNR, and lowest RMSE for iteration number, the highest SDNR for VM-energy, mini-batch size, epochs, and standard deviation of domain filter were selected as optimum parameters.

Fig. 5

Assessing improvements in image quality using the signal-difference-to-noise ratio (SDNR), a statistical model with a Gumbel distribution, and the radial-modulation transfer function (radial-MTF) of the selected features. (a) The in-focus plane image shows the simulated ground-glass opacity (GGO) nodule and background areas of the SDNR, (b) measurements and the ripple artifact with simulated GGO nodule areas of the Gumbel analysis, and (c) measurements of the simulated GGO nodule areas of the radial-MTF.

Evaluation of image quality

SDNR was compared between DE-VM-VDSR and the conventional algorithms (polychromatic; FBP, SART, SART-TV-FISTA, DE-VM-SART-TV-FISTA) to assess the increase in simulated nodule contrast on each in-focus plane image. We further ascertained that a Gumbel distribution [47] and radial-modulation transfer function (radial-MTF) [48] are statistical models that can be utilized to determine the influence of ripple artifacts and spatial resolution of a simulated GGO nodule on the features of images in the in-focus plane. Comparison with the DE-VM-VDSR algorithm was conducted by choosing an effective algorithm for simulated nodule contrast, ripple artifact, and spatial resolution of lesion polychromatic (120 kV) FBP, SART, AND SART-TV-FISTA, and DE-VM-SART-TV-FISTA (energy is determined by optimization). DE-VM-VDSR was evaluated using the application image generated by using the optimization parameters.

Statistical model with a Gumbel distribution

Gumbel distribution is a method used to quantitatively analyze streak artifacts of high-frequency components found in cross-sectional images [47]. High-frequency artifacts in DT images have been quantitatively analyzed using Gumbel distribution [34]. Therefore, we think that employing this statistical model, we can quantitatively represent the high-frequency ripple artifacts on DT images. The analysis procedure is shown below.

(1) A rectangular window with a width of 24 pixels and a length (X-ray sweep direction) of 24 pixels was placed on each in-focus plane image nearly perpendicular to multiple streak artifacts, as shown in Fig 5 (b).

(2) The parallel-line profiles of pixel values at 1-pixel intervals [as shown by the arrow in Fig 5 (b)] were determined to yield a total of 23 parallel-line pixel-value profiles (each sampling size: 23).

(3) The pixel-value profiles were graphed, and the maximal variations between adjacent pixel values were determined and analyzed employing a Gumbel distribution.

(4) Cumulative probability function was measured employing the symmetry rank method [cumulative probability $= \frac{(\partial - 0.5)}{\ell}$, $(\text{for } \partial = 1, \ldots \ell)$; ℓ is the sampling size] with order statistics.

(5) Generating the probability diagram, the maximal difference between adjacent pixels shows linearity with the cumulative probability; thus, the Gumbel characteristic can be calculated with the maximal difference between neighboring pixels.

Finally, to evaluate linearity, Pearson's correlation coefficient was determined and examined [probability (P) values < 0.01] using SPSS Statistics for Windows [version 24.0 (SPSS Inc., Chicago, IL, USA)].

Radial MTF

The radial MTF [48] considers that the edge of a circular object is a combination of all edges on a line extending in the radial direction from the center of the circle, and acquires a plurality of profiles in the radial direction from the center of the object. The edge-spread function is obtained by averaging the pixel-value profile of the edge image, the line-spread function is obtained from the difference, and the one-dimensional fast Fourier transform is used to calculate the MTF. The spatial resolution of simulated GGO nodule was calculated in the radial direction including the BG field from the center of the nodule as the evaluation area (radius: 9 pixels, area: 254 pixels) [Fig 5(c)].

Results

After measuring the RMSE of each iteration, the optimal number of iterations (k, m) in IR for SART and SART-TV-FISTA converged to 30 [Fig 6 (a)]. Therefore, the number of iterations was set to 30. Because 280 keV showed the highest SDNR in the VM-energy optimization of DE-VM-SART-TV-FISTA, the energy in DE-VM processing was selected to be 280 keV [Fig 6 (b)]. Next, to verify optimization with a mini-batch size of 64, 128, 256, and 512, the DSNR were measured with epochs of 5, 10, 20, 30, and 40. For epochs of 20 and a mini-batch size of 256, the SDNR value was the highest [Fig 6 (c)]. Therefore, learning was conducted by setting the mini-batch size to 256 and epochs to 20 (hyper-parameter). To optimize the domain filter (σ_d) standard deviation of bilateral filtering, SDNR was measured by changing σ_d to 1, 2, and 3, the highest SDNR was 1 [Fig 6 (d)]. Therefore, σ_d was set to 1. Using the results of the optimization verification,

DE-VM-VDSR images were generated by setting the number of iterations (k , m) to 30, mini-batch size to 256, epochs to 20, and standard deviation of domain filter (σ_d) to 1, and the simulated nodule contrast (SDNR), ripple artifact (Gumbel distribution), and spatial resolution (radial MTF) were evaluated and compared with the images from conventional algorithms.

Fig. 6

Optimization results for parameter [number of iterations for iterative reconstruction (IR), dual-energy virtual monochromatic (DE-VM) energy, mini-batch, epochs, and standard deviation of domain filter σ_d] determination. (a) The root-mean square error (RMSE) with respect to numbers of iteration are shown for each polychromatic IR algorithm. (b) The signal-difference-to-noise ratio (SDNR) resulting from differences in the DE-VM energy in the simultaneous algebraic reconstruction technique (SART) total variation (TV) first iterative shrinkage-thresholding algorithm (FISTA) [DE-VM-SART-TV-FISTA] are shown. (c)The SDNR resulting from differences in the mini-batch and epochs in the DE-VM with very-deep super-resolution (VDSR) reconstruction algorithm (DE-VM-VDSR) are shown. (d) The SDNR resulting from differences in the σ_d in the DE-VM-VDSR are shown. From the results of the optimization verification, DE-VM-VDSR images were generated by setting the number of iterations to 30, mini-batch size to 256, epochs to 20, and σ_d to 1.

Fig 7 reveals the recreated images of the chest phantom N1 with a simulated GGO nodule obtained using the DE-VM-VDSR algorithm or conventional reconstruction algorithms. Remarkably, DT images generated by the DE-VM-VDSR algorithm showed decreased ripple artifacts and increased simulated nodule contrast; specifically, the edges on the simulated nodule were sharpened and more clearly identified. In contrast, images produced with polychromatic FBP exhibited more noise and ripple artifacts, and

polychromatic SART, SART-TV-FISTA, and DE-VM-SART-TV-FISTA exhibited reduced sharpness and contrast in the simulated nodule.

Fig. 7

Comparisons among the dual-energy (DE) virtual monochromatic (VM) with very-deep super-resolution (VDSR) reconstruction algorithm (DE-VM-VDSR) and conventional reconstruction algorithms (DE-VM-VDSR { 0 – 0.23 }, FBP { kernel: Ramp; 0 – 0.4 }, SART { 120 kV; 0 – 0.4 }, SART-TV-FISTA { 120 kV; 0 – 0.4 }, DE-VM-SART-TV-FISTA { 280 keV; 0 – 0.23 }) in the in-focus plane. The range of the chest phantom N1 with lung-field display are varied to compare contrast and background gray levels. For each corresponding set, VM (DE-VM-VDSR, and DE-VM-SART-TV-FISTA) images are displayed at the same window width and level, whereas the polychromatic FBP and IR images have larger window widths because the backgrounds are less flattened. The X-ray source is moved vertically along the image.

Abbreviations: FBP = filtered back projection, SART = simultaneous algebraic reconstruction technique, TV-FISTA = total variation first iterative shrinkage-thresholding algorithm, IR = iterative reconstruction.

Fig 8 shows the SDNR results for the ROI set on the chest NI phantom. DE-VM-VDSR yielded the highest simulated GGO nodule contrast regardless of the conventional algorithm (SDNR; 0.9454). For the polychromatic imaging algorithms, FBP, SART, and SART-TV-FISTA, and DE-VM-SART-TV-FISTA, simulated GGO nodule contrast relied on the type of reconstruction algorithm (SDNR; FBP = 0.6582, SART = 0.4272, SART-TV-FISTA = 0.6410, DE-VM-SART-TV-FISTA = 0.6838).

Fig. 8

Plots of the SDNR versus each algorithms from the in-focus plane. Comparisons of the signal-difference-to-noise ratio (SDNR) of in-focus plane images obtained via the dual-energy (DE) virtual

monochromatic (VM) with very-deep super-resolution (VDSR) reconstruction algorithm (DE-VM-VDSR) and the conventional reconstruction algorithms.

Fig. 9 is a Gumbel plot of the relationships between the maximal variations and predicted cumulative probabilities (DE-VM-VDSR 0.0077 ± 0.0004, FBP 0.0198 ± 0.0013, SART 0.0130 ± 0.0006, SART-TV-FISTA 0.0099 ± 0.0005, SART-TV-FISTA with VM 0.0102 ± 0.0005). Here, the largest variations are distributed linearly (DE-VM-VDSR r = 0.910 [$P < 0.01$]; polychromatic FBP r = 0.971 [$P < 0.01$]; polychromatic SART r = 0.918 [$P < 0.01$]; polychromatic SART-TV-FISTA r = 0.943 [$P < 0.01$]; DE-VM-SART-TV-FISTA r = 0.944 [$P < 0.01$]). These observations verified the use of the Gumbel distribution as an acceptable statistical model for defining the largest variations in the differences between nearby pixel-value profiles. Additionally, analyzing the maximal variations in the Gumbel plot recognize DE-VM-VDSR as yielding the lowest high-frequency ripple artifacts. The polychromatic SART-TV-FISTA algorithms exhibited less high-frequency ripple artifacts than DE-VM-SART-TV-FISTA, whereas the polychromatic FBP algorithm distribution differed from other algorithms.

Fig. 9

The largest variations extracted from 23 pixel-value profiles are plotted. Relatively large variations in pixel value were attributed to high-frequency ripple artifacts.

The spatial resolution of the simulated GGO nodule region is shown in Fig 10. DE-VM-VDSR showed the highest spatial resolution. In particular, the improvement in the low frequency region (~ 0.05 cycles / mm) was remarkable. For polychromatic SART and DE-VM-SART-TV-FISTA, the spatial resolution tended to decrease. Among the

conventional algorithms, polychromatic FBP showed similar spatial resolution to DE-VM-VDSR.

Fig. 10

Plots of the radial-modulation transfer function (radial-MTF) versus spatial frequency (cycles/mm) from the in-focus plane for the dual-energy (DE) virtual monochromatic (VM) with very-deep super-resolution (VDSR) reconstruction algorithm (DE-VM-VDSR) and conventional reconstruction algorithms. Spatial frequency of simulated ground-glass opacity (GGO) nodule area indicates the simulated GGO nodule and background areas of the radial-MTF measurements. The plots from the area of the GGO nodule and background regions.

Discussion

The newly established DE-VM-VDSR algorithm, that combines DE-VM and VDSR for projection space processing with SART-TV-FISTA prior to reconstruction with bilateral filtering, produced adequate overall functioning in this study. This hybrid algorithm generated better image quality of the simulated GGO nodule, present in the chest phantom. Furthermore, this algorithm effectively lowered ripple artifacts from the images, specifically at bigger distances from the ribs. Notably, the DE-VM-VDSR was principally useful for enhancing pulmonary GGO nodule contrast. In summary, this algorithm may be a new option that has promising applications for chest imaging because it produced far superior images than those processed employing conventional algorithms with respect to contrast and ripple artifacts. On the basis of the chest imaging conditions and required characteristics of the final images, DE-VM-VDSR provides flexibility in selecting imaging parameters that may be helpful to users.

The VM method can be employed to produce material-selective images that are devoid of beam hardening artifacts. As the mass density of each basis material at each point in the image is dependent on the linear attenuation coefficients of the basis materials at a nominal photon energy, beam hardening artifacts can lead to erroneous mass densities of the basis materials [34]. Thus, we think that our new DE-VM-VDSR, which is created by the combination of the VM-VDSR processing and SART-TV-FISTA after reconstruction of bilateral filtering algorithms, could effectively enhance contrast and lower ripple artifacts (Fig 8, 9).

VDSR is a training strategy that makes it possible to recreate an image at any scale. Thus, a hybrid training strategy is helpful for SR images. A well-trained network can process images of all scales and reconstruct the input image to any size. VDSR recreates the residual image, making it simpler to examine the difference between LR and HR [49]. The VDSR generated edge and texture are made sharper. Residual learning is capable of estimating the residuals between LR and SR images so that LR images can be utilized to create final SR images. DE-VM-VDSR images as can be visulaized by this feature in the radial MTF plot (Fig 10).

In CNN-based approaches, the upscaling process can use transposed convolution (so-called fractionally strided convolutional) layers [50, 51] or sub-pixel layers [52]. On the other hand, the weights of these trained networks are set for a specific scale factor [53]. This is a restrictive feature of CNN-based SR for DT projection data, since a fixed upscaling factor is not ideal in this scenario. This is because using fixed upscaling, high-frequency image content cannot be retrieved from the low-resolution image. We have presented a multiscale CNN-based SR method for three-dimensional CDT that is able to learn multiple scales by training multiple scale factors employing an independent

upscaling method, for example, bicubic interpolation.

The following two factors are critical in attaining outstanding functioning of the CNN-based SR algorithm: selecting the procedures of evaluating enhancements in image quality (contrast and spatial resolution) and generating adequate training data. Choosing the appropriate image quality enhancing method dictates if sufficient data is provided for the CNN-based SR to differentiate simulated GGO nodules from normal structures. The training data for CNN-based SR comprises as many types of pulmonary GGO nodules as possible.

In FISTA, the initial value of the next iteration is evaluated by a linear combination of the results of the two earlier iterations. Bearing in mind that GGO nodules are dispersed inside specific lung fields, the reconstruction of CDT images is normally scarce with some local high-frequency regions. This suggests that a combination of regularizations that potentiate the conditions of sparsity and smoothness could additionally enhance the recreation of CDT. The most basic sparse index is the L_0 norm, which is the number of non-zero elements in all vectors. However, the normal inverse problem of L_0, at least in the undetermined case, is difficult to solve [54]. In contrast, L_1 norm is a convex relaxation of the L_0 norm and frequently employed to improve the sparsity of images, specifically in the field of CS [45, 55]. Optimization difficulties can be resolved by many algorithms such as gradient projection [56], iterative shrinkage-thresholding [57], and FISTA [43, 44], etc. We trust that FISTA is ideal as it can assess fast and precise L_1 solutions that can solve the reconstruction problem and improve the sparsity of CDT images.

TV-minimization assumes that a true image is comparatively uniform and piece

wise. Since TV is defined as the sum of the first-order derivative magnitudes [58], noise and artifacts show as valleys and peaks and have relatively larger TV values. Thus, TV is restricted to clinical CT. The combination of the CNN-based SR algorithm and FISTA-TV has the ability to learn nonlinear regression and effectively use prior knowledge to future pulmonary GGO nodules and CDT images.

Preferably, structures in each plane of interest must be displayed clearly in the corresponding plane of DT reconstruction, while those present outside of the plane should be rendered invisible [59]. Fundamentally, the restricted angular range of the DT image acquisition geometry restricts the spatial resolution in the dimension perpendicular to the detector plane [59]. Because of this, out-of-plane structures cannot be totally reduced from the reconstruction plane and thus are always present. But, majority of these structures are invisible as different low-amplitude structures from projections overlap in the reconstruction plane, and cause the blurring of image. To the contrary, out-of-plane structures from high-attenuation features cannot be lowered and show-up as replicates in each reconstruction plane, excepting in the plane where the actual high-attenuation feature (dorsal lib) is present. At one projection angle, these ghosting features (ripple artifact) are spread along the line made between the actual feature and the X-ray source. DE-VM-VDSR algorithm has been beneficial for lowering ripple artifacts but may remove needed clinical features.

Our DE-VM-VDSR algorithm has some limitations. First, even though, we have chosen the three-material decomposition method to ascertain numerical stability, further perfections in this area are required. The precision of three-material decomposition is restricted on current DT systems, specifically for the elements with low fractions [60]. Second, the indicated learning model is proposed for a specific type of phantom with

simulated pulmonary nodules and may not work efficiently when the trained network is adjusted to projection data correction from scanning geometries that are entirely different. Future studies are needed to design a learning model that can be employed in general cases. Finally, DE acquisition is a mechanical constraint, and not many devices are able to quickly switch tube voltages during a single acquisition.

Conclusion

In this chest phantom study, we compared the newly developed DE-VM-VDSR and various conventional DT reconstruction algorithms without and with VM processing. Particularly, our DE-VM-VDSR increased contrast and reduced high-frequency ripple artifacts and generated relatively better results in terms of improvement of spatial resolution when compared with conventional reconstruction algorithms.

Acknowledgments

We wish to thank Mr. Kazuaki Suwa and Yuuki Watanabe at Department of Radiology Dokkyo Medical University Koshigaya Hospital for support on experiment.

This study was supported by a grant from Kitasato University School of Allied Health Sciences (Grant-in-Aid for Research Project, No. 2020-1006).

References

1. Yankelevitz DF, Reeves AP, Kostis WJ, Zhao B, Henschke CI. Small pulmonary nodules: volumetrically determined growth rates based on CT evaluation. Radiology.

2000;217(1):251-6. Epub 2000/09/30. doi: 10.1148/radiology.217.1.r00oc33251. PubMed PMID: 11012453.

2. Stitik FP, Tockman MS. Radiographic screening in the early detection of lung cancer. Radiol Clin North Am. 1978;16(3):347-66.

3. Muhm JR, Miller WE, Fontana RS, Sanderson DR, Uhlenhopp MA. Lung cancer detected during a screening program using four-month chest radiographs. Radiology. 1983;148(3):609-15. Epub 1983/09/01. doi: 10.1148/radiology.148.3.6308709. PubMed PMID: 6308709.

4. Siegelman SS, Khouri NF, Leo FP, Fishman EK, Braverman RM, Zerhouni EA. Solitary pulmonary nodules: CT assessment. Radiology. 1986;160(2):307-12. Epub 1986/08/01. doi: 10.1148/radiology.160.2.3726105. PubMed PMID: 3726105.

5. Bray F, Ferlay J, Soerjomataram I, Siegel RL, Torre LA, Jemal A. Global cancer statistics 2018: GLOBOCAN estimates of incidence and mortality worldwide for 36 cancers in 185 countries. CA Cancer J Clin. 2018;68(6):394-424. Epub 2018/09/13. doi: 10.3322/caac.21492. PubMed PMID: 30207593.

6. Zwirewich CV, Vedal S, Miller RR, Muller NL. Solitary pulmonary nodule: high-resolution CT and radiologic-pathologic correlation. Radiology. 1991;179(2):469-76. Epub 1991/05/01. doi: 10.1148/radiology.179.2.2014294. PubMed PMID: 2014294.

7. Murata K, Itoh H, Todo G, Kanaoka M, Noma S, Itoh T, et al. Centrilobular lesions of the lung: demonstration by high-resolution CT and pathologic correlation. Radiology. 1986;161(3):641-5. Epub 1986/12/01. doi: 10.1148/radiology.161.3.3786710. PubMed PMID: 3786710.

8. Miller ER, McCurry EM, Hruska B. An infinite number of laminagrams from a finite number of radiographs. Radiology. 1971;98(2):249-55. Epub 1971/02/01. doi: 10.1148/98.2.249. PubMed PMID: 5541045.

9. Baily NA, Lasser EC, Crepeau RL. Electrofluoroplanigraphy. Radiology. 1973;107(3):669-71. Epub 1973/06/01. doi: 10.1148/107.3.669. PubMed PMID: 4702547.

10. Kruger RA, Nelson JA, Ghosh-Roy D, Miller FJ, Anderson RE, Liu PY. Dynamic tomographic digital subtraction angiography using temporal filtration. Radiology. 1983;147(3):863-7. Epub 1983/06/01. doi: 10.1148/radiology.147.3.6342037. PubMed PMID: 6342037.

11. Sone S, Kasuga T, Sakai F, Aoki J, Izuno I, Tanizaki Y, et al. Development of a high-resolution digital tomosynthesis system and its clinical application. Radiographics. 1991;11(5):807-22. Epub 1991/09/01. doi: 10.1148/radiographics.11.5.1947318. PubMed PMID: 1947318.

12. Sabol JM. A Monte Carlo estimation of effective dose in chest tomosynthesis. Med Phys. 2009;36(12):5480-7. Epub 2010/01/26. doi: 10.1118/1.3250907. PubMed PMID: 20095260.

13. Dobbins JT, 3rd, Godfrey DJ. Digital x-ray tomosynthesis: current state of the art and clinical potential. Phys Med Biol. 2003;48(19):R65-106. Epub 2003/10/29. PubMed PMID: 14579853.

14. Gomi T, Nakajima M, Fujiwara H, Umeda T. Comparison of chest dual-energy subtraction digital tomosynthesis imaging and dual-energy subtraction radiography to detect simulated pulmonary nodules with and without calcifications a phantom study. Acad Radiol. 2011;18(2):191-6. Epub 2011/01/15. doi: 10.1016/j.acra.2010.09.021. PubMed PMID: 21232683.

15. Gomi T, Nozaki M, Takeda T, Umeda T, Takahashi K, Nakajima M. Comparison of chest dual-energy subtraction digital tomosynthesis and dual-energy subtraction radiography for detection of pulmonary nodules: initial evaluations in human clinical cases. Acad Radiol. 2013;20(11):1357-63. Epub 2013/10/15. doi: 10.1016/j.acra.2013.08.006. PubMed PMID: 24119347.

16. Machida H, Yuhara T, Mori T, Ueno E, Moribe Y, Sabol JM. Optimizing parameters for flat-panel detector digital tomosynthesis. Radiographics. 2010;30(2):549-

62. Epub 2010/03/17. doi: 10.1148/rg.302095097. PubMed PMID: 20228334.

17. Ma G, He T, Yu Y, Duan H, Yang C. Improving Image Quality of Bronchial Arteries with Virtual Monochromatic Spectral CT Images. PLoS One. 2016;11(3):e0150985. Epub 2016/03/12. doi: 10.1371/journal.pone.0150985. PubMed PMID: 26967737; PubMed Central PMCID: PMCPMC4788436.

18. Otrakji A, Digumarthy SR, Lo Gullo R, Flores EJ, Shepard JA, Kalra MK. Dual-Energy CT: Spectrum of Thoracic Abnormalities. Radiographics. 2016;36(1):38-52. Epub 2016/01/14. doi: 10.1148/rg.2016150081. PubMed PMID: 26761530.

19. Chae EJ, Song JW, Seo JB, Krauss B, Jang YM, Song KS. Clinical utility of dual-energy CT in the evaluation of solitary pulmonary nodules: initial experience. Radiology. 2008;249(2):671-81. Epub 2008/09/18. doi: 10.1148/radiol.2492071956. PubMed PMID: 18796658.

20. Alvarez RE, Macovski A. Energy-selective reconstructions in X-ray computerized tomography. Phys Med Biol. 1976;21(5):733-44. Epub 1976/09/01. PubMed PMID: 967922.

21. Kalender WA, Perman WH, Vetter JR, Klotz E. Evaluation of a prototype dual-energy computed tomographic apparatus. I. Phantom studies. Med Phys. 1986;13(3):334-9. Epub 1986/05/01. doi: 10.1118/1.595958. PubMed PMID: 3724693.

22. Zhao W, Vernekohl D, Han F, Han B, Peng H, Yang Y, et al. A unified material decomposition framework for quantitative dual- and triple-energy CT imaging. Med Phys. 2018;45(7):2964-77. Epub 2018/04/22. doi: 10.1002/mp.12933. PubMed PMID: 29679500.

23. Cann CE, Gamsu G, Birnberg FA, Webb WR. Quantification of calcium in solitary pulmonary nodules using single- and dual-energy CT. Radiology. 1982;145(2):493-6. Epub 1982/11/01. doi: 10.1148/radiology.145.2.7134457. PubMed PMID: 7134457.

24. Zhang K, Zuo W, Chen Y, Meng D, Zhang L. Beyond a gauaaian denoiser: residual learning of deep CNN for image denoising. IEEE Trans Image Process. 2017;26(7):3142 - 55.

25. Chen H, Zhang Y, Kalra MK, Lin F, Chen Y, Liao P, et al. Low-Dose CT With a Residual Encoder-Decoder Convolutional Neural Network. IEEE Trans Med Imaging. 2017;36(12):2524-35. Epub 2017/06/18. doi: 10.1109/TMI.2017.2715284. PubMed PMID: 28622671; PubMed Central PMCID: PMCPMC5727581.

26. Wolterink JM, Leiner T, Viergever MA, Isgum I. Generative Adversarial Networks for Noise Reduction in Low-Dose CT. IEEE Trans Med Imaging. 2017;36(12):2536-45. Epub 2017/06/03. doi: 10.1109/TMI.2017.2708987. PubMed

PMID: 28574346.

27. Yoon Y, Jeon HG, Yoo D, Lee JY, Kweon IS. Learning a deep convolutional network for light-field image super-resolution IEEE international conference on computer vision workshop. 2015:57 - 65.

28. Park J, Hwang D, Kim KY, Kang SK, Kim YK, Lee JS. Computed tomography super-resolution using deep convolutional neural network. Phys Med Biol. 2018;63(14):145011. Epub 2018/06/21. doi: 10.1088/1361-6560/aacdd4. PubMed PMID: 29923839.

29. Umehara K, Ota J, Ishida T. Application of Super-Resolution Convolutional Neural Network for Enhancing Image Resolution in Chest CT. J Digit Imaging. 2018;31(4):441-50. Epub 2017/10/20. doi: 10.1007/s10278-017-0033-z. PubMed PMID: 29047035; PubMed Central PMCID: PMCPMC6113156.

30. Gu Y, Lu X, Yang L, Zhang B, Yu D, Zhao Y, et al. Automatic lung nodule detection using a 3D deep convolutional neural network combined with a multi-scale prediction strategy in chest CTs. Comput Biol Med. 2018;103:220-31. Epub 2018/11/06. doi: 10.1016/j.compbiomed.2018.10.011. PubMed PMID: 30390571.

31. Hamidian S, Sahiner B, Petrick N, Pezeshk A. 3D Convolutional Neural Network for Automatic Detection of Lung Nodules in Chest CT. Proc SPIE Int Soc Opt

Eng. 2017;10134. Epub 2017/08/29. doi: 10.1117/12.2255795. PubMed PMID: 28845077; PubMed Central PMCID: PMCPMC5568782.

32. Halder A, Dey D, Sadhu AK. Lung Nodule Detection from Feature Engineering to Deep Learning in Thoracic CT Images: a Comprehensive Review. J Digit Imaging. 2020. Epub 2020/01/31. doi: 10.1007/s10278-020-00320-6. PubMed PMID: 31997045.

33. kim J, Lee JK, Lee KM. Accurate Image Super-Resolution Using Very Deep Convolutional Networks. 2016 IEEE Conference on Computer Vision and Pattern Recognition (CVPR). 2016. doi: 10.1109/CVPR.2016.182.

34. Gomi T, Sakai R, Goto M, Hara H, Watanabe Y. Development of a novel algorithm for metal artifact reduction in digital tomosynthesis using projection-based dual-energy material decomposition for arthroplasty: A phantom study. Phys Med. 2018;53:4-16. Epub 2018/09/23. doi: 10.1016/j.ejmp.2018.07.011. PubMed PMID: 30241753.

35. Berger M, Hubbell J. Photon cross sections on a personal computer. Gent Radiat Res. 1987:1-28. doi: 10.2172/6016002.

36. Krizhevsky A, Sutskever I, Hinton GE. Imagenet classification with deep convolutional neural networks. Advances in neural information processing systems. 2012:1097 - 105.

37. Sutskever I, Martens J, Dahl G, Hinton G. On the importance of initialization and momentum in deep learning. Proceedings of the 30th international conference on machine learning. 2013;PMLR 28(3):1139 - 47.

38. Andersen AH, Kak AC. Simultaneous algebraic reconstruction technique (SART): a superior implementation of the art algorithm. Ultrason Imaging. 1984;6(1):81-94. Epub 1984/01/01. doi: 10.1177/016173468400600107. PubMed PMID: 6548059.

39. Xu S, Lu J, Zhou O, Chen Y. Statistical iterative reconstruction to improve image quality for digital breast tomosynthesis. Med Phys. 2015;42(9):5377-90. Epub 2015/09/04. doi: 10.1118/1.4928603. PubMed PMID: 26328987; PubMed Central PMCID: PMCPMC4545099.

40. Lu Y, Chan HP, Wei J, Hadjiiski LM, Samala RK. Multiscale bilateral filtering for improving image quality in digital breast tomosynthesis. Med Phys. 2015;42(1):182-95. Epub 2015/01/08. doi: 10.1118/1.4903283. PubMed PMID: 25563259; PubMed Central PMCID: PMCPMC4277560.

41. Choi S, Lee H, Lee D, Choi S, Lee CL, Kwon W, et al. Development of a chest digital tomosynthesis R/F system and implementation of low-dose GPU-accelerated compressed sensing (CS) image reconstruction. Med Phys. 2018;45(5):1871-88. Epub 2018/03/04. doi: 10.1002/mp.12843. PubMed PMID: 29500855.

42. Miyata K, Nagatani Y, Ikeda M, Takahashi M, Nitta N, Matsuo S, et al. A phantom study for ground-glass nodule detectability using chest digital tomosynthesis with iterative reconstruction algorithm by ten observers: association with radiation dose and nodular characteristics. Br J Radiol. 2017;90(1071):20160555. Epub 2017/01/20. doi: 10.1259/bjr.20160555. PubMed PMID: 28102693; PubMed Central PMCID: PMCPMC5601533.

43. Beck A, Teboulle M. A Fast Iterative Shrinkage-Thresholding Algorithm for Linear Inverse Problems. SIAM Journal on Imaging Sciences. 2009;2(1):183-202. doi: 10.1137/080716542.

44. Beck A, Teboulle M. Fast gradient-based algorithms for constrained total variation image denoising and deblurring problems. IEEE Trans Image Process. 2009;18(11):2419-34. Epub 2009/07/29. doi: 10.1109/TIP.2009.2028250. PubMed PMID: 19635705.

45. Donoho DL. Compressed sensing. IEEE Transactions on Information Theory. 2006;52(4):1289-306. doi: 10.1109/tit.2006.871582.

46. Sidky EY, Pan X. Image reconstruction in circular cone-beam computed tomography by constrained, total-variation minimization. Phys Med Biol. 2008;53(17):4777-807. Epub 2008/08/15. doi: 10.1088/0031-9155/53/17/021. PubMed

PMID: 18701771; PubMed Central PMCID: PMCPMC2630711.

47. Imai K, Ikeda M, Enchi Y, Niimi T. Statistical characteristics of streak artifacts on CT images: relationship between streak artifacts and mA s values. Med Phys. 2009;36(2):492-9. Epub 2009/03/19. doi: 10.1118/1.3056554. PubMed PMID: 19291988.

48. Richard S, Husarik DB, Yadava G, Murphy SN, Samei E. Towards task-based assessment of CT performance: system and object MTF across different reconstruction algorithms. Med Phys. 2012;39(7):4115-22. Epub 2012/07/27. doi: 10.1118/1.4725171. PubMed PMID: 22830744.

49. Zhang H, Wang P, Zhang C, Jiang Z. A Comparable Study of CNN-Based Single Image Super-Resolution for Space-Based Imaging Sensors. Sensors (Basel). 2019;19(14). Epub 2019/07/26. doi: 10.3390/s19143234. PubMed PMID: 31340511; PubMed Central PMCID: PMCPMC6679528.

50. Ozan O, Wenjia B, Matthew L, Ricardo G, Konstantinos K, Jose C, et al. Multi-input Cardiac Image Super-Resolution Using Convolutional Neural Networks. Medical Image Computing and Computer-Assisted Intervention - MICCAI 2016. 2016:246-54. doi: 10.1007/978-3-319-46726-9_29.

51. Chao D, Chen CL, Xiaoou T. Accelerating the Super-Resolution Convolutional Neural Network. Computer Vision – ECCV 2016. 2016:391-407. doi: 10.1007%2F978-

3-319-46475-6_25.

52. Wenzhe S, Jose C, Ferenc H, Johannes T, Andrew PA, Rob B, et al. Real-Time Single Image and Video Super-Resolution Using an Efficient Sub-Pixel Convolutional Neural Network. 2016 IEEE Conference on Computer Vision and Pattern Recognition (CVPR). 2016:1874-83. doi: 10.1109/CVPR.2016.207.

53. Pham CH, Tor-Diez C, Meunier H, Bednarek N, Fablet R, Passat N, et al. Multiscale brain MRI super-resolution using deep 3D convolutional networks. Comput Med Imaging Graph. 2019;77:101647. Epub 2019/09/08. doi: 10.1016/j.compmedimag.2019.101647. PubMed PMID: 31493703.

54. Natarajan BK. Sparse Approximate Solutions to Linear Systems. SIAM Journal on Computing. 1995;24(2):227-34. doi: 10.1137/s0097539792240406.

55. Candes EJ, Wakin MB. An Introduction To Compressive Sampling. IEEE Signal Processing Magazine. 2008;25(2):21-30. doi: 10.1109/msp.2007.914731.

56. Figueiredo MAT, Nowak RD, Wright SJ. Gradient Projection for Sparse Reconstruction: Application to Compressed Sensing and Other Inverse Problems. IEEE Journal of Selected Topics in Signal Processing. 2007;1(4):586-97. doi: 10.1109/jstsp.2007.910281.

57. Daubechies I, Defrise M, De Mol C. An iterative thresholding algorithm for

linear inverse problems with a sparsity constraint. Communications on Pure and Applied Mathematics. 2004;57(11):1413-57. doi: 10.1002/cpa.20042.

58. Du Y, Wang X, Xiang X, Wei Z. Evaluation of hybrid SART + OS + TV iterative reconstruction algorithm for optical-CT gel dosimeter imaging. Phys Med Biol. 2016;61(24):8425-39. Epub 2016/11/16. doi: 10.1088/0031-9155/61/24/8425. PubMed PMID: 27845916.

59. Wu T, Moore RH, Kopans DB. Voting strategy for artifact reduction in digital breast tomosynthesis. Med Phys. 2006;33(7):2461-71. Epub 2006/08/11. doi: 10.1118/1.2207127. PubMed PMID: 16898449.

60. Liu X, Yu L, Primak AN, McCollough CH. Quantitative imaging of element composition and mass fraction using dual-energy CT: three-material decomposition. Med Phys. 2009;36(5):1602-9. Epub 2009/06/24. doi: 10.1118/1.3097632. PubMed PMID: 19544776; PubMed Central PMCID: PMCPMC2719492.

Figure_1

Click here to access/download;Figure;Fig1.tif

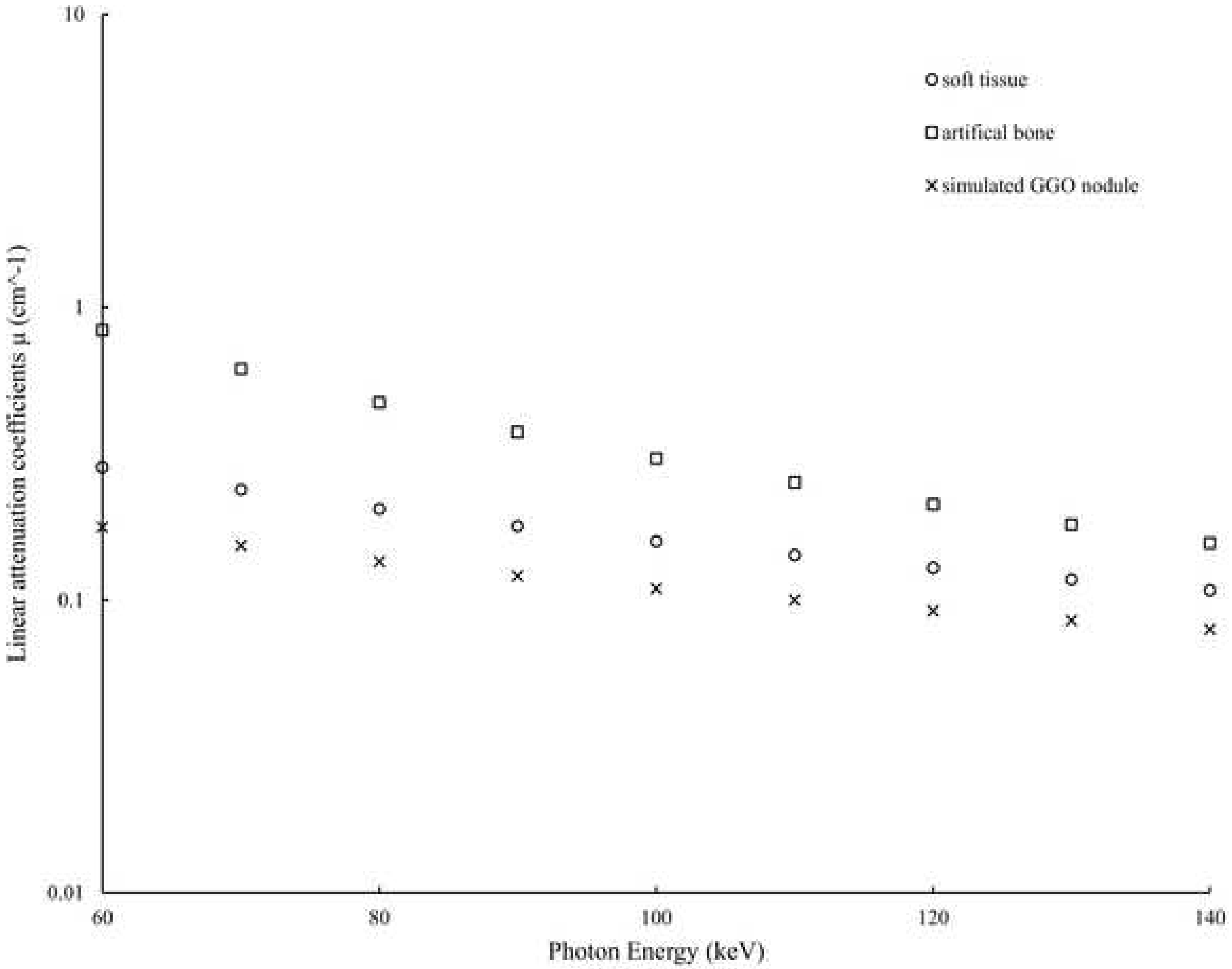

Figure_2

Click here to access/download;Figure;Fig2.tif

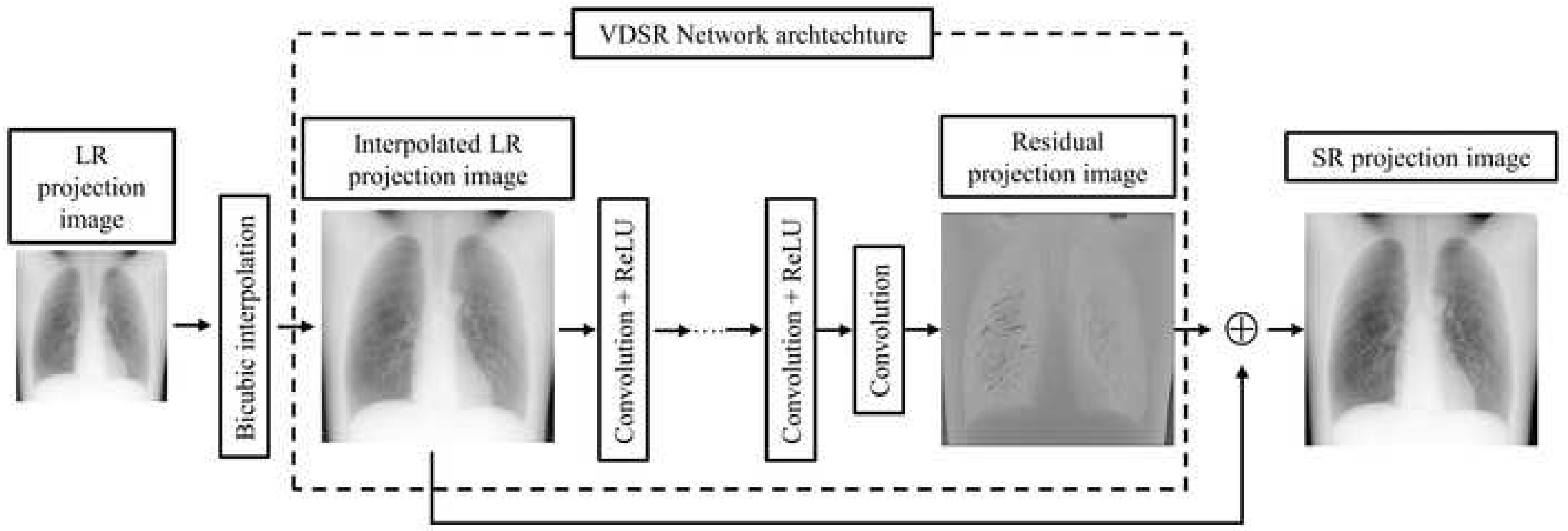

Figure_3

Click here to access/download;Figure;Fig3.tif

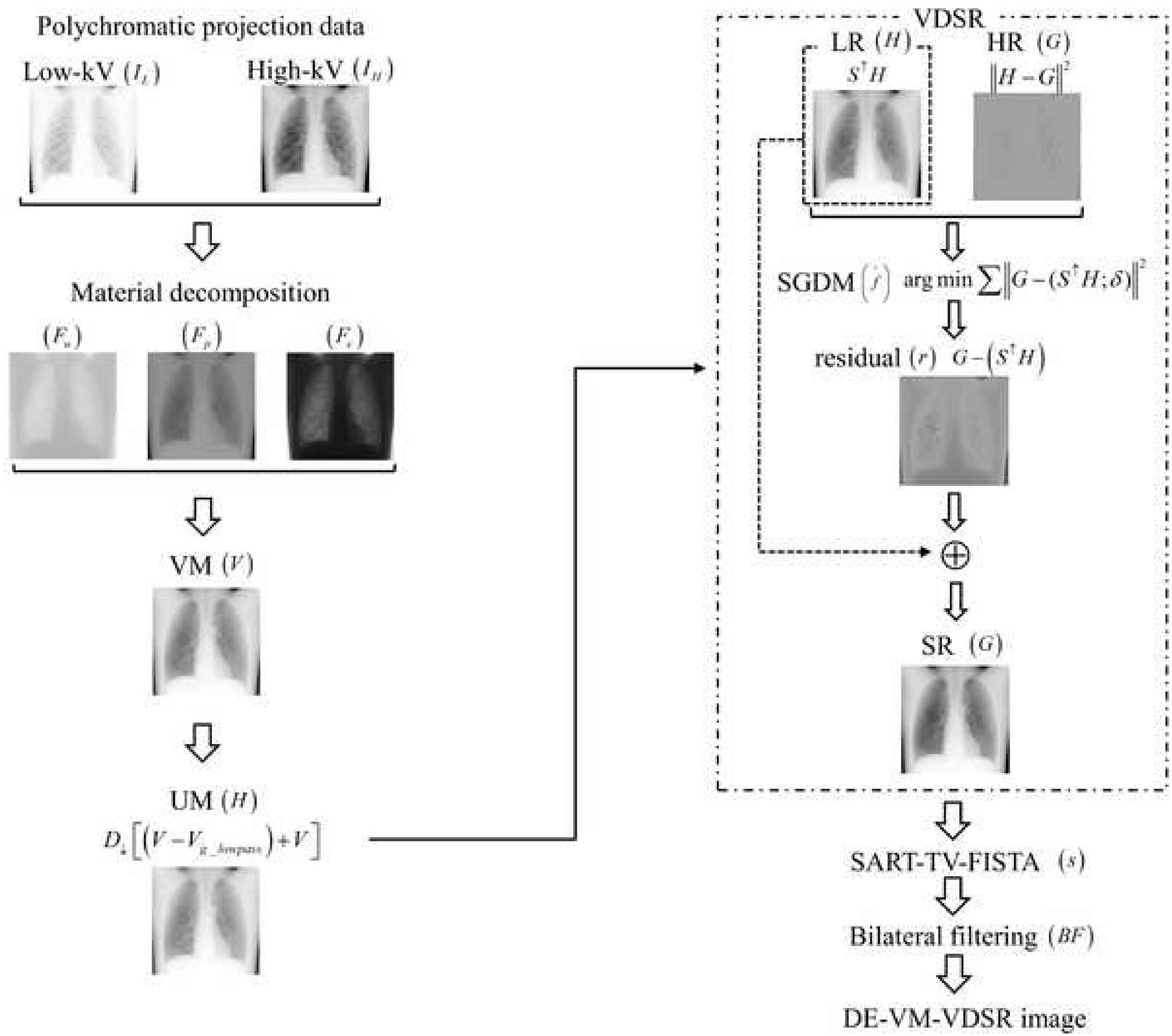

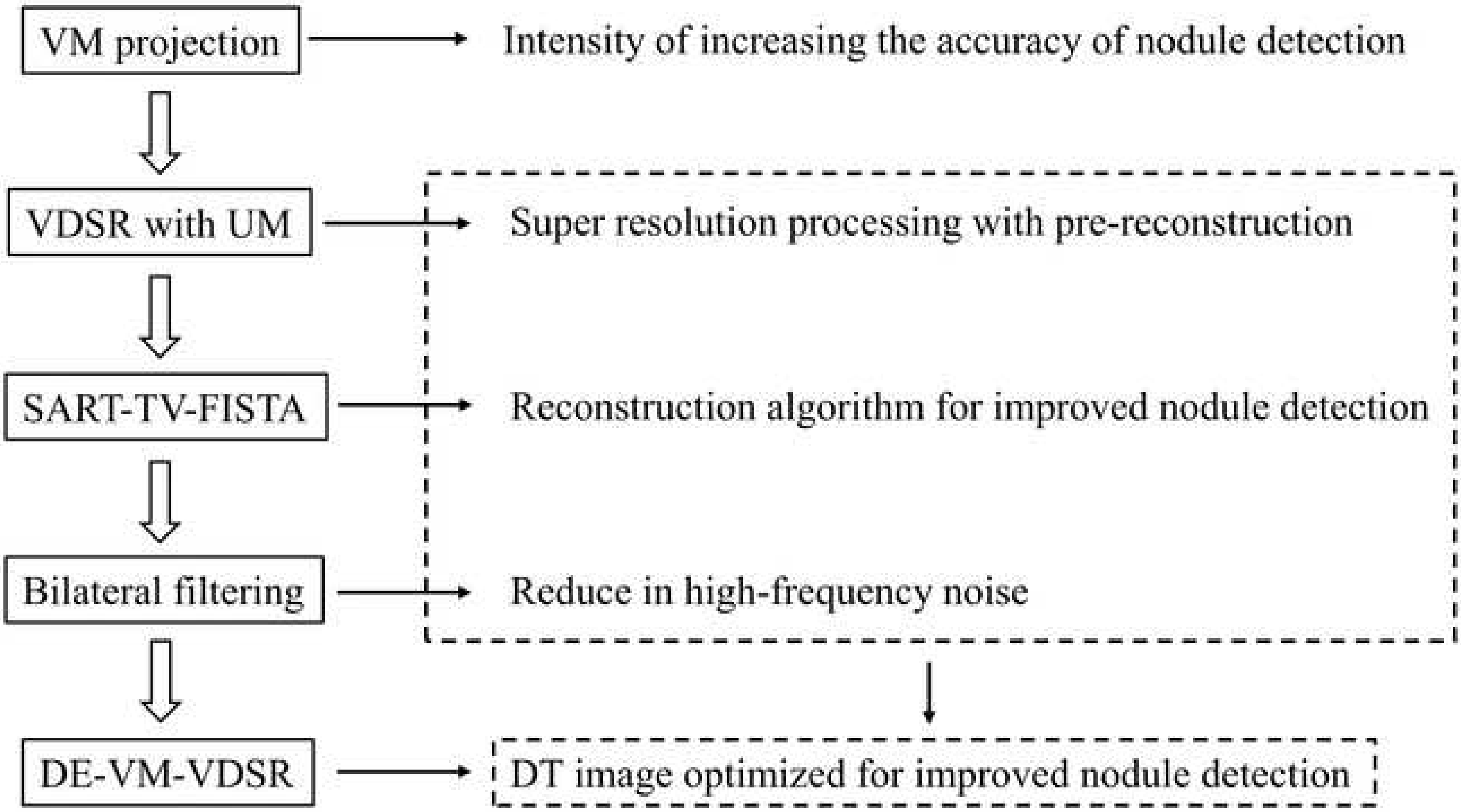
VM projection
Intensity of increasing the accuracy of nodule detection
VDSR with UM
Super resolution processing with pre-reconstruction
SART-TV-FISTA
Reconstruction algorithm for improved nodule detection
Bilateral filtering
Reduce in high-frequency noise
DE-VM-VDSR
DT image optimized for improved nodule detection

Figure_5

Click here to access/download;Figure;Fig5.tif

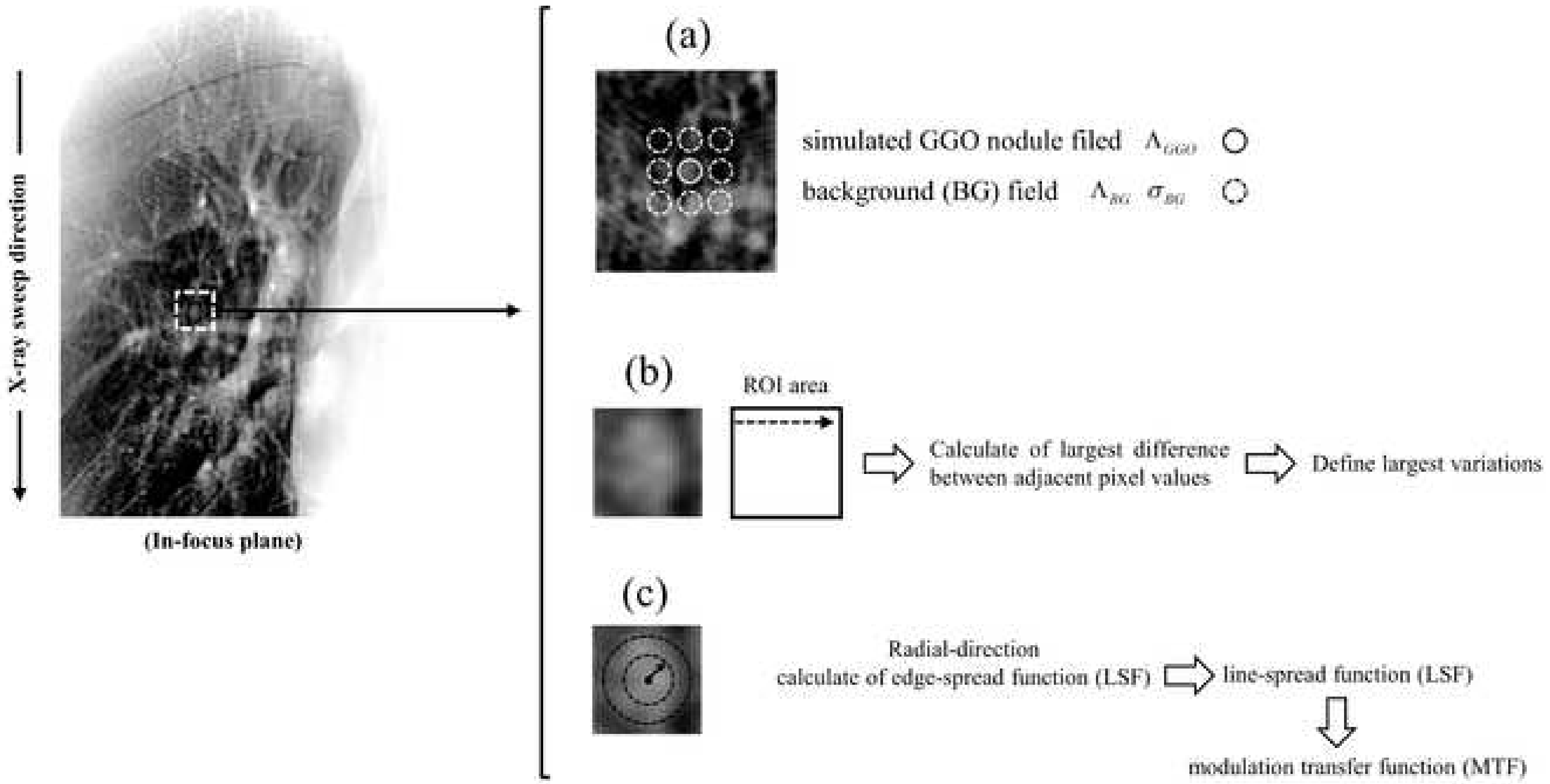

Figure_6

Click here to access/download;Figure;Fig6.tif

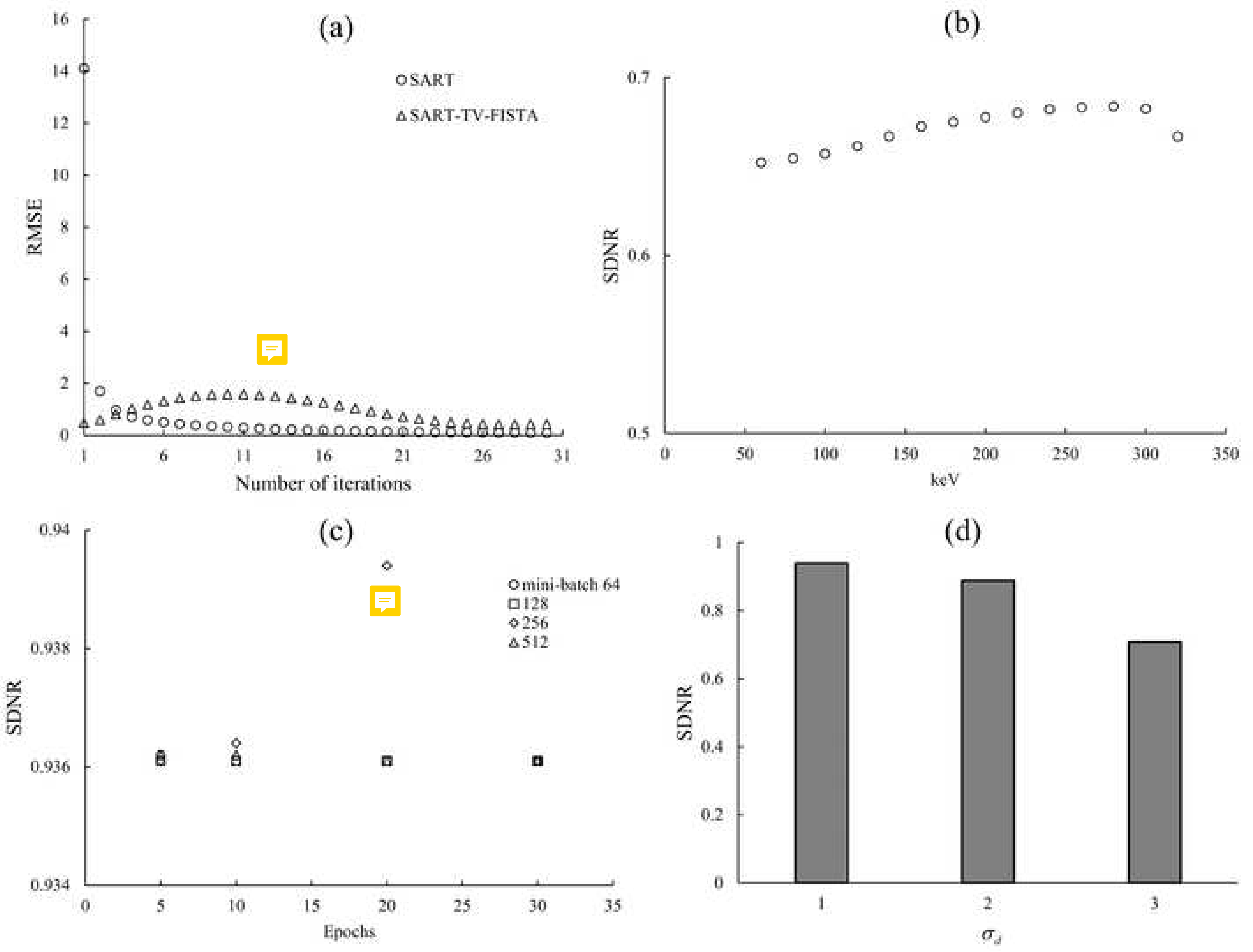

Figure_7

Click here to access/download;Figure;Fig7.tif

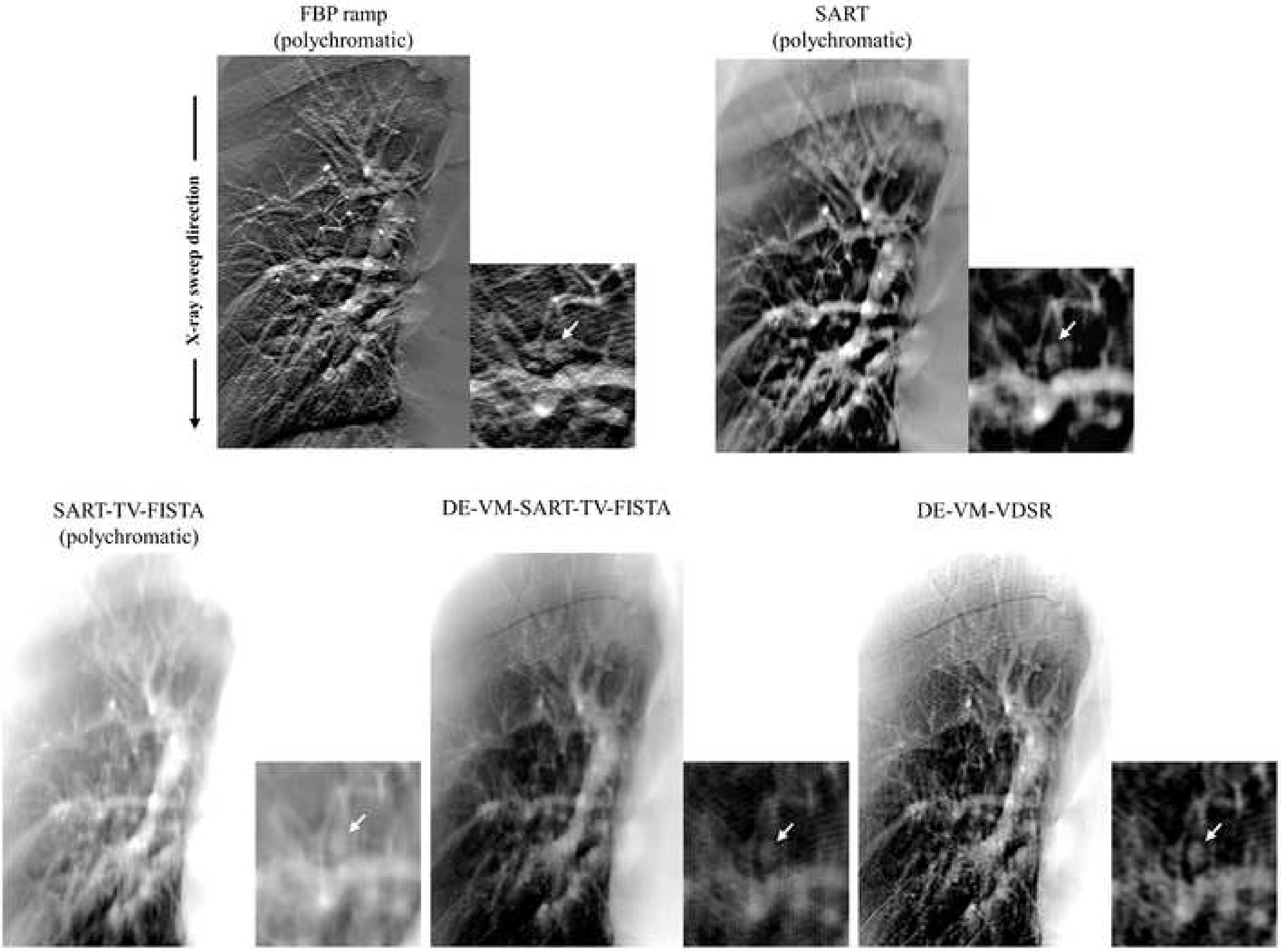

Figure_8

Click here to access/download;Figure;Fig8.tif

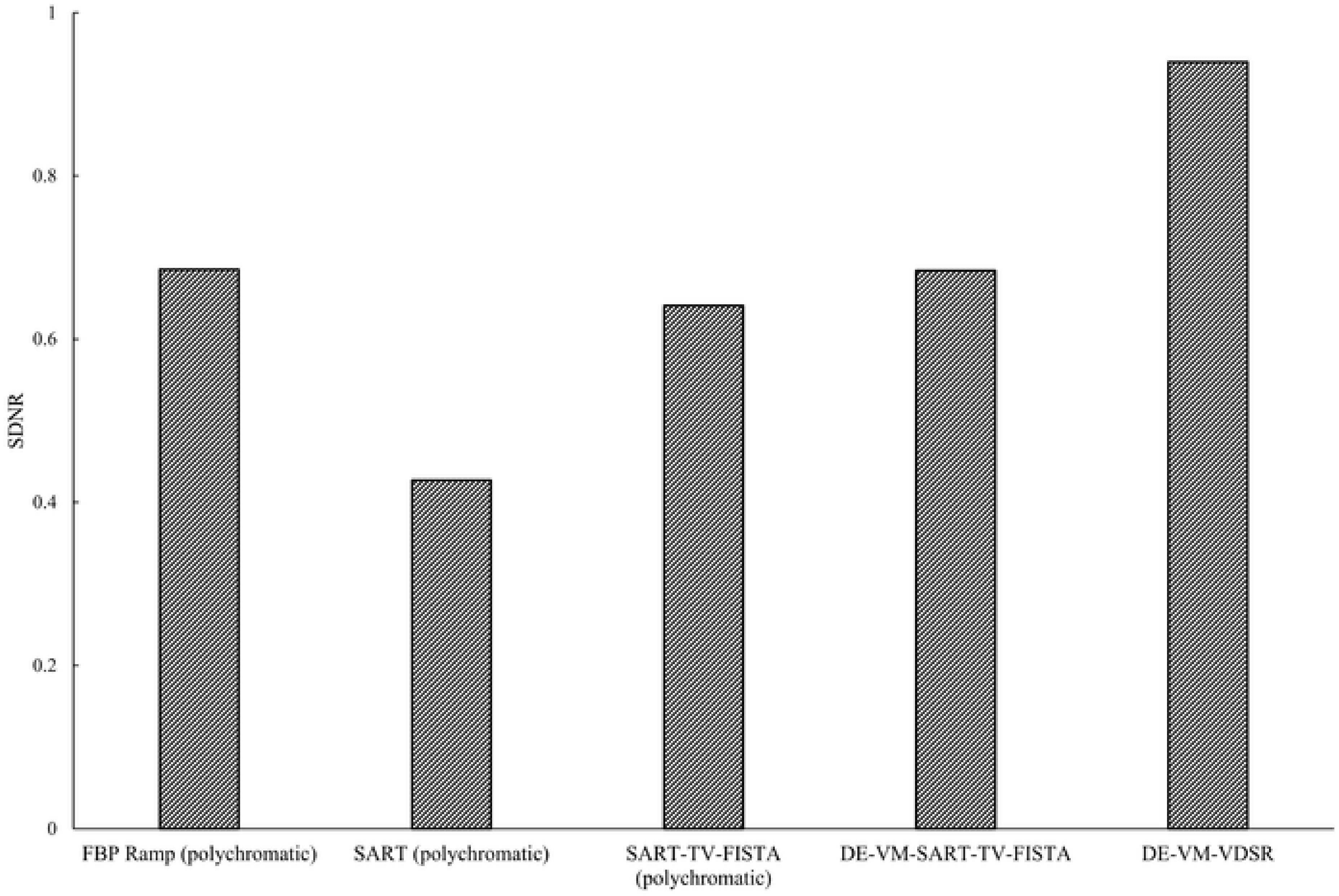

Figure_9

Click here to access/download;Figure;Fig9.tif

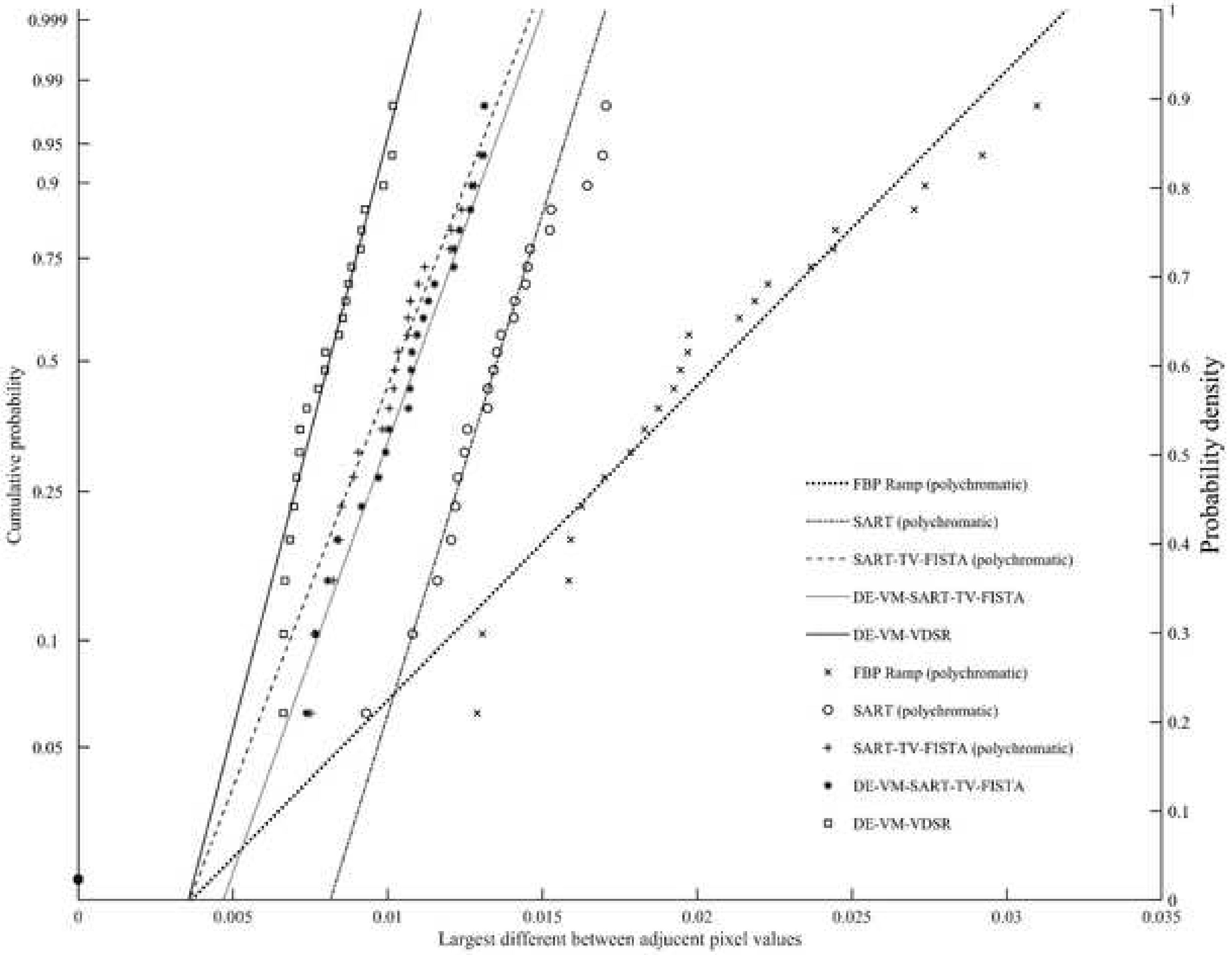

Figure_10

Click here to access/download;Figure;Fig10.tif

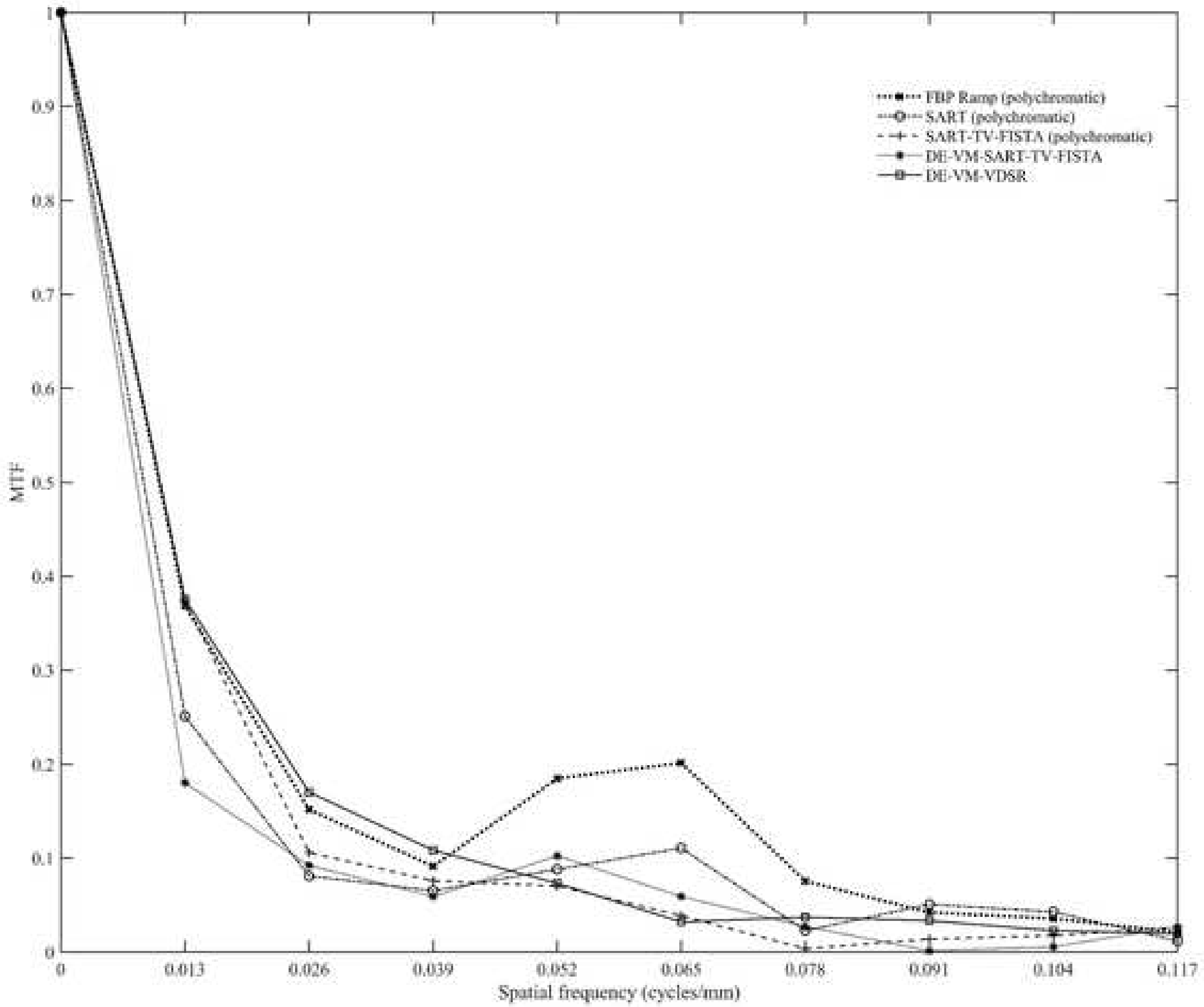

Supplement: Attachment — Submitted filename: PONE-D-20-19441_reviewer-comment_responce.pdf [file pone.0244745.s003.pdf]
