# Supplementary material for: Improved digital chest tomosynthesis image quality by use of a projection-based dual-energy virtual monochromatic convolutional neural network with super resolution
Source: PLoS One. 2020 Dec 31;15(12):e0244745. doi: 10.1371/journal.pone.0244745 (PMC7774945; doi:10.1371/journal.pone.0244745)
Supplement: Attachment — Submitted filename: rebuttal_letter_2.pdf [file pone.0244745.s004.pdf]

MS Number: PONE-D-20-19441R1

MS Title: Improved digital chest tomosynthesis image quality by use of a projection-based dual-energy virtual monochromatic convolutional neural network with super resolution

PLOS ONE Academic Editor

Dear Dr. Jeonghwan Gwak

Thank you in advance for your kind consideration of this paper. I attach here our revised manuscript, as well as a point-by-point response to the reviewer's comments.

We feel that the 2th revised manuscript is a suitable response to the comments, and is significantly improved over the 1th revised submission.

Sincerely yours.

Response to the comments of Reviewer #1:

We wish to express our appreciation to the reviewer for offering insightful comments, which have helped us improve the paper significantly.

Comment: It seems that the ground truth (HR) projections in training the VDSR network is the original CDT acquisitions. If this is the case, the importance of the whole work would be dramatically decreased. Since the LR projections are all artificially down sampled, the clinical importance of the proposed workflow would land on obtaining low resolution projections to reduce imaging noise and time. In addition, all the result images should be compared with the reconstruction from the original projections with just the VDSR being excluded. The authors should add comments on this.

Response: We selected conventional algorithms (polychromatic FBP, SART, SART-TV-FISTA, DE-VM-SART-TV-FISTA) as comparison targets for the VE-VM-VDSR with BF algorithms and evaluated their performance. The images and data shown in the results are the results of the conventional algorithms reconstructed from the original projections. Corrected the description of conventional algorithms.

[Evaluation of image quality]

> SDNR was compared between DE–VM–VDSR with and without BF and the conventional algorithms (polychromatic FBP, SART, SART-TV-FISTA, DE-

VM-SART-TV-FISTA with reconstruction from the original projections) to assess the increase in simulated nodule contrast on each in-focus plane image.

(Remark 1)

Also, as the reviewers have pointed out, there are limits to the benefits that can be gained from this workflow, so we added it to the discussion.

[Discussion]

HR projections in training the VDSR network is the original CDT acquisitions. Because all the LR projections are artificially downsampled, obtaining LR projections is an important step in the proposed workflow to reduce the imaging noise and processing time.

(Remark 2)

Comment: The total acquisition time is 6.4s. Then if we want to clinically apply this workflow, the patients should hold their breath for such a time. Some comments about the challenges about the anticipated clinical application should also be added.

Response: Accordingly, we have added “the issue of CDT acquisition time” to discussion section.

[Discussion]

Because CDT acquisition requires an imaging time of 6.4 s, it may be difficult to hold breath depending on the patient’s condition when assuming clinical use. Currently, patients whose condition is stable will be considered for examination. We hope that the acquisition time can be reduced by improving the specifications of the acquisition system (detector sensitivity, hardware control, etc.).

(Remark 3)

Comment: According to Fig. 8(c), the combinations of 256/10 and 512/40 have the highest SDNR, why did you choose 128/70?

Response: According to Kim et al. optimization verification [(evaluated by peak signal-to-noise ratio (PSNR)], epoch changes to around 40, but tends to be constant (converged) after 40. With reference to these results, the highest value of epoch & mini-batch showing a convergence tendency was selected as the optimization. We have added “reasons for choosing optimized epoch & mini-batch” to Optimization parameters section.

* kim J, Lee JK, Lee KM. Accurate Image Super-Resolution Using Very Deep Convolutional Networks. 2016 IEEE Conference on Computer Vision and Pattern Recognition (CVPR). 2016. doi: 10.1109/CVPR.2016.182.

[Optimization parameters]

According to the optimization verification conducted by Kim et al. (evaluated by peak signal-to-noise ratio (PSNR), epoch changes to around 40 but tends to be constant (converge) after 40. With reference to these results, the highest values of epoch and mini-batch indicating a tendency of convergence were selected as the optimization values.

We have added "reasons for choosing optimized epoch & mini-batch" to Optimization parameters section.

(Remark 4)

Comment: The Introduction seems still lengthy and need to be logically improved. Please try to re-read it and make some revision.

Response: In accordance with the reviewer's request. We have shorted the revised manuscript. We wish to thank the reviewer for this comment.

Comment: Some grammar issues still can be found, such as lines 464-466: "NPS acquired uniform water phantom … and analyzed them by using the two-dimensional Fourier analysis method [54] using an in-focus plane."

Response: We have had the manuscript rewritten by an experienced scientific editor, who has improve the grammar and stylistic expression of the paper.

Thank you again for your comments on our paper. I trust that the revised manuscript is now suitable for publication.
